# Valuing impact: Estimating return on investment of mental health and wellbeing projects for veterans and first responders

**DOI:** 10.1371/journal.pone.0353179

**Published:** 2026-08-05

**Authors:** Itismita Mohanty, Theo Niyonsenga, Luis Salvador-Carulla, Cindy Woods, Sue Lukersmith

**Affiliations:** Health Research Institute, Faculty of Health, University of Canberra, University Drive Bruce, ACT 2601, Canberra, Australia; Taipei Medical University, TAIWAN

## Abstract

**Background:**

Veterans and first responders like firefighters, police officers, paramedics, and military personnel face higher rates of mental health issues and suicide compared to the general population. Implementing and evaluating mental health projects for Veterans and first responders is crucial for enhancing their ability to serve society effectively. Economic evidence is increasingly used to improve the effectiveness and accountability of health and social programs. However, current methods are insufficient for comparing resource efficiency and value for money across different sectors and countries. This study introduces a novel methodology to accommodate heterogeneity across multiple projects within a grant program, enable standardization, and facilitate comparison in real-world implementation research.

**Methods:**

The choice of perspective in economic evaluation depends on context, stakeholder viewpoints, resource availability, and intended use. We used a flexible approach, combining various methods to standardize resource utilization, valuation, and outcome assessment. This allowed us to estimate an overall return on investment at the program level by pooling diverse projects together. We evaluated costs from the funder's perspective, using general population valuation for healthcare interventions.

**Results:**

We estimated overall and individual project Return on Investment ratios for the funder's investment in the Mental Health Program, considering both total and in-kind costs. Results show that for every $1 invested in the Veterans and first responders Mental Health Program, the program returns $1.14 in overall health value, slightly exceeding the financial resources invested.

**Conclusion:**

Standard economic evaluation methodologies do not accommodate real-world complexities and the heterogeneity across projects inherent in grant programs. Yet the development of pragmatic economic methods to assess value and return on investment of grant programs is essential given current funding pressures. Our study demonstrates the feasibility of addressing economic evaluations across heterogenous, international projects. Although not strictly adhering to established methods, our pragmatic approach focuses on funder return on investment. This methodology provides a pathway for economic analysis of grant programs.

## 1. Introduction

Veterans and first responders (VFRs), including firefighters, police officers, paramedics, and military personnel, are often exposed to highly stressful and traumatic events in their line of duty [1,2]. While their roles are critical in protecting communities and ensuring public safety, these responsibilities place them at an increased risk for mental health challenges [1–3]. Veterans, for instance, may struggle with the lasting psychological impact of combat and transitioning to civilian life. Similarly, first responders often encounter critical incidents, including severe injuries, fatalities, and disasters, which may lead to accumulated stress and burnout. Studies consistently show that prolonged exposure to trauma, the nature of their work, and a culture that may stigmatize seeking help contribute to mental-ill health [1,3].

The common mental health issues that VFRs experience include post-traumatic stress disorder (PTSD), depression, anxiety, and alcohol or substance abuse [4–6]. These mental health challenges not only affect the VFRs, but it also extends to their families, workplaces, and communities [7]. Untreated health issues can lead to job loss, strained relationships, and a heightened risk of suicide. Research suggests that veterans and first responders experience suicide rates significantly higher than the general population [8,9], underscoring the urgent need for effective mental health and wellbeing interventions.

Investing in programs aimed at mental health support and resilience-building for VFRs not only improves individual outcomes but also enhances their capacity to continue serving society effectively [10]. Evaluating the return on such investments is crucial to ensure the sustainability and scaling of impactful programs, as it helps funders and policymakers understand their economic and social value.

Few evaluation methods effectively address the heterogeneity and complexity inherent in answering key questions of real-world implementation research, such as “What matters?”, “What is value-based care?” and “What is the value for money or return on investment, for whom, and how?” while comparing across multiple and highly heterogeneous projects [11]. Economic considerations play a pivotal role in answering these questions, as healthcare funders are hesitant to invest in ongoing implementation strategy support without clear evidence of return on their investment [11]. Conducting comparative economic evaluations of implementation strategies is crucial to providing payers, policymakers, and providers with the necessary insights to determine whether specific strategies represent an efficient use of limited organizational resources. However, while economic evaluations in health services are relatively common, those focused on implementation remain in their early stages [11]. Also, it is not yet a common practice to collect cost data during the implementation process, further very few perform comparative economic analyses of different implementation strategies, even when these strategies target similar (though not identical) health outcomes. Understanding the costs and translating the outcomes of implementation strategies are essential for determining the costs and resources needed to scale up and spread effective practices [11–13].

In this context, while specified methods are designed to support the uptake of effective practices in implementation studies, strictly adhering to the guidelines of a specific economic evaluation framework often appears impractical due to methodological rigidities and the complexities of real-world implementation research. These challenges are even more pronounced when attempting comparative economic evaluations across a range of heterogenous projects, given the heterogeneity in project settings, resources and outcome measures used.

## 2. Study context

The economic evaluation reported in this manuscript was one component of an ecosystem approach used in the evaluation and impact analysis of a significant international grant program, detailed elsewhere [14] but briefly described here. In 2021, the funding body (philanthropic organisations with a focus on men’s health), launched an international grant Program focused on addressing mental health, wellbeing, and suicide prevention among Veterans and First Responders. The Veterans and First Responders (VFR) grant Program targeted the funding agency’s key markets (Australia, Canada, Ireland, Germany, New Zealand, United Kingdom, and the United States). Overall, the VFR Grant Program funded 15 diverse preventive and/or early intervention mental health promotion initiatives (hereafter referred to as “Projects”) targeting either veterans, first responders, their families, carers, or associated organizations across the seven countries. Subsequently, the funding agency also, engaged external researchers (the authors) to conduct a comprehensive third-party evaluation of the Grant Program from November 2021 to October 2024. In line with the funding agency’s objectives and its commitment to measure impacts beyond individual outcomes, the scope of the evaluation extended beyond documenting mental health outcomes to understand not only **whether** improvements occurred, but also **how**, **why**, and under what **conditions** these improvements can be maintained, replicated, or expanded in a cost‑effective way. The evaluation used multiple indicators across the domains across the logic for change (resource-throughputs-output) refined through an expert panel. Indicator examples include implementation process domains, context, Project characteristics (such as genealogy, mechanisms, communication mode) and participant mental health outcomes. A range of different data collection and analysis methods were used to identify which Projects are effective, sustainable, scalable, and represent value for money as outlined in the published protocol [14,15].

## 3. Economic evaluation in implementation studies

Economic evaluation aims to quantify the costs and outcomes of interventions, with the broader aim of informing efficient resource allocation. Specific methods that are used in implementation and intervention research are cost-effectiveness analysis (CEA), cost-utility analysis (CUA), cost-benefit analysis (CBA), budget impact analysis and comparative effectiveness [16–18]. Recently Return on Investment analysis (ROI) has been in the forefront due to its methodological flexibility to mixed method research for real-world applicability [19–21]. Crawley-stout et al. have described ROI as a performance measure used to evaluate investment efficiency in financial terms [22]. Banke-Thomas et al. [23] have advocated for the use of the societal perspective of ROI or Social Return on Investment (SROI) across healthcare. Thusini et al [24] analyse various ways ROI is conceptualized and applied in large-scale healthcare quality improvement programs, integrating economic and non-economic perspectives. They identify that ROI in healthcare Quality Improvement often aligns with the broader concepts of value and benefit, encompassing both monetary and non-monetary outcomes. The review highlights the importance of considering diverse stakeholder perspectives such as managerial, healthcare staff, patient or society. However, there are considerable challenges on how health outcomes are measured, valued and the unit of analysis used, for example, patient-level in cost-effectiveness analysis and country-level in budget impact analysis. Other challenges include inadequate skills for ROI evaluation, lack of credible financial proxies, a lack of consensus on; who to include as beneficiaries, how to account for counterfactual and appropriate study-time horizon [23,24].

CEA and CUA estimate the cost to achieve specific health outcomes. CEA compares resources used with health benefits gained, like depression-free days. CUA, a broader variant, uses health outcomes as a composite measure of mortality and morbidity, typically expressed as quality-adjusted life years (QALYs) [25]. In implementation studies, cost-effectiveness analysis offers flexibility to accommodate implementation-specific outcomes. However, its cost-effectiveness ratios may not be directly comparable across studies. Both methods require setting a threshold to determine cost-effectiveness, which can be challenging for specific outcomes. CBA, on the other hand, compares the monetary value of resources used with the monetary value of outcomes, but assigning monetary values to health outcomes is complex and controversial. It's useful when outcomes can be reasonably monetized. CBA forms the basis for ROI and SROI; CBA and SROI adopt a societal perspective, while ROI is focused on managerial and investor perspective [23,24].

Return on Investment (ROI) estimates the costs and benefits from a program, expressed as an ROI metric. This methodology converts (monetises) costs and benefits into ROI. ROI is reported as metric (percentage or a ratio), e.g., ROI = 1:1 means a 100% return was made. In finance, ROI can be as basic as dollars out versus dollars in, but in healthcare, ROI can also be medical costs avoided, reduced absenteeism, improved health outcomes or access to care, increased engagement, or positive experiences [24]. Thusini et al. [24] provide a conceptual framework for understanding ROI in healthcare quality improvement programs. Nonetheless, it is important to gain deeper understanding into how ROI is calculated in the healthcare industry, the challenges associated with these calculations, and best practices for measuring the effectiveness of healthcare investments. SROI, in particular, is measured from the perspective of society as a whole. SROI is useful for assessing community-based or non-profit projects, as it engages stakeholders in defining and valuing project impacts and offers a way to assign proxy monetary values to both objective and subjective outcomes. This framework enhances transparency and accountability, helping to prioritize actions that maximize the social value of a project [26].

However, SROI's reliance on subjective assumptions, stakeholder input, and proxy values can be very context-specific, which may be helpful for capturing detailed and in-depth impacts at the individual Project level. Nonetheless, the lack of a standard framework in valuing health outcomes and measuring resource use may introduce project-specific bias. Additionally, monetizing non-market outcomes may oversimplify complex social impacts, making it challenging to compare these impacts across multiple projects, such as in the overall evaluation of the VFR Program.

In this paper, we conduct an economic analysis of the ROI for the funder as part of a broader impact analysis of the 2021 VFR Program with final data collected from each project by March 2024. As a secondary aim we demonstrate the usability of FROI as part of impact analysis of funding programs and projects.

## 4. Method

### 4.1. The overall VFR program and projects evaluation

As discussed earlier, this research forms part of a broader, multi-layered evaluation of an international philanthropic funding organization – funded VFR Mental Health Program. We monitored and evaluated the Program from two perspectives: 1) the individual funded Projects (n = 15), and 2) The VFR grant Program overall. The expected outcomes of the VFR evaluation were to identify Projects or strategies that are: acceptable, feasible and effective; have a positive impact on veterans, first responders and their families; provide value for money or return on investment for the funder. The evaluation assessed the Projects and the overall Program performance across five domains: Funder Return on Investment (FROI), Complexity, Implementation Processes, Outcomes and Performance. The domains, indicators and selected outcome measures were developed through rigorous analytical methods, a co-design process, and sustained engagement with the funder and all Project teams, with the full protocol [14] and implementation process evaluation presented elsewhere [15].

In this paper we focus on the methods and results of the return on investment analysis for the funder, which constitutes one domain of the overall evaluation. As such, we propose the term *Funder Return on Investment* (FROI) to more accurately reflect this targeted scope. We have estimated FROI ratios at both the Project and overall Program levels. The FROI framework allows us to assess the direct value generated from the funder's investment, distinct from broader social impacts, offering a tailored insight into the specific return on resources dedicated by the funding agency. As Sittimart et al [27] highlighted identifying the perspective in health economic analysis is crucial because it underscores the necessary variations in methodological recommendations and definitions. The application of these perspectives defines the scope of costs and outcomes considered, thereby influencing the study's conclusions and recommendations. There are two measurements of Funder Return on Investment (FROI), one at the funding agency overall VFR mental health Program level and other one at the individual Project level. In each case (Program and Project levels), we estimated two separate FROI ratios. The first used the funding provided by the awarded agency only. However, at individual Project level, the grant funding typically does not cover all resource costs. Therefore, a second FROI calculation was performed which included the awarded agency funding plus in-kind costs and other financial support the Project received. All models and methodologies are non-proprietary and fully disclosed in the manuscript to ensure transparency and reproducibility.

#### 4.1.1. The source and availability of the data.

As independent external evaluators of the VFR Grant Program, we accessed de-identified data through formal confidentiality agreements with each participating organization and their participants in the seven countries. Data cannot be shared due to ethical and legal obligations. While we were granted permission to analyse these data, we do not hold rights to share them publicly or with journals. Each organization conducted its own data collection and administered outcome measures, such as questionnaires. De-identified datasets were securely transferred via AARNet FileSender and stored on password-protected, firewall-secured servers. The data were accessed between December 2022 and April 2023 for research purposes.

Ethical approval was received on the 18 February 2022 by the University of Canberra Human Research Ethics Committee #9346. To comply with General Data Protection Regulation (GDPR) and other privacy requirements, Projects developed participant information and consent forms stating that de-identified data would be shared with the evaluation team. Participants confirmed they had read and understood this and provided explicit consent as a separate item, with the option to withdraw at any time. All participants consented accordingly, and the external evaluation team reviewed blank consent forms from each project to ensure compliance. Any data access requests should be directed to the University of Canberra Human Research Ethics Committee (HumanEthicsCommittee@canberra.edu.au).

#### 4.1.2. Costs measurement.

We estimated all the costs associated with the Projects in consultation with the respective Project leads and the funding body, including both monetary and non-monetary costs. This includes direct costs such as Program/Project expenses, as well as indirect costs such as additional staff time or volunteer hours.

#### 4.1.3. Outcome measurement.

For estimating the FROI ratio, at the overall Grant Program level and to facilitate comparison across the Projects within the program, it is important to assign a monetary value to the mental health outcomes achieved by the different projects. There were 120 patient reported outcome measures used across the 15 projects for different mental health conditions. The five common mental health and wellbeing indicators identified were depression and anxiety, trauma‑related stress, resilience, support, and quality of life. For these indicators, across the projects there were 39 different outcome measures used. The next step was to identify the most used outcome measure per indicator to determine a ‘common comparator’. Once established, an external expert panel was engaged and underwent a critical review process using a decision tree approach to determine whether outcome measures were concurrent or convergent with the indicator’s common comparator. The review process was supported by a preceding extensive search and analysis of peer reviewed literature to extract papers and relevant papers on concurrency and convergence. The results of this search were presented to the expert panel for discussion and consensus on concurrency or convergence and are published elsewhere [15]. By identifying five common indicators for evaluation and selecting a “common comparator” for each indicator, the evaluation team and expert panel were able to assess 39 different outcome measures and enable cross‑Project comparisons across all five indicators, despite variability in Project aims, measurement tools, and feasibility constraints [14].

Health-Related Quality of Life (HRQoL) and Quality Adjusted Life Years (QALY) are the outcome measures used in health economic evaluations [25,28]. QALYs are used to quantify improvements in health outcomes, assessed on a generic preference based HRQoL scale such as EQ5D-5L or EQ5D-3L, attributable to the project [28]. This is done by combining the utility values generated from the HRQoL quality of life scale with survival time to provide a summary measure of health outcomes over a specified period. Changes in QALYs resulting from the project can therefore be used to express its overall quality‑of‑life impact in monetary terms.

However, in this evaluation we used utility values (average annualised adjustments) rather than the change in QALY to enable comparison across heterogeneous Projects. Utility values are preference‑based numerical weights that reflect the desirability of different health states [29,30]. They quantify health‑related quality of life (HRQoL) on a scale anchored at:1 = full health, 0 = death and < 0 = health states considered worse than death (possible in some value sets). These values capture both morbidity and functional limitations and allow HRQoL to be expressed on a common, cardinal scale suitable for economic evaluation [29,30]. Individuals report their health across multiple dimensions to define a specific health state profile. These health states are then assigned utility values based on societal preferences derived from population‑based studies, using valuation methods such as time trade‑off, standard gamble, or discrete choice experiments.

HRQoL is a multi-dimensional concept that encompasses the physical, mental, emotional, functional, and social aspects of a person's well-being that are influenced by their health [31]. It is an important measure used to understand how individuals perceive their overall health status and how it impacts their ability to live fulfilling lives. Even so, generic preference-based HRQoL measures that can be used to estimate utility values were not included in most of the VFR Projects as condition-specific measures are considered more informative in capturing the Project specific outcome. We selected the Patient Health Questionnaire-9 (PHQ-9) [32] and the Generalised Anxiety Disorder-7 (GAD-7) [33] as they were identified as the common comparators for the depression and anxiety domain, but also because they are widely used, condition‑specific symptom measures that could be translated into the broader Health Related Quality of Life (HRQol) utilities for economic evaluation [34]. These measures assess a patient’s mental health but are not designed to inform utility values. Mapping between the condition-specific measure and a generic preference-based HRQoL measure using regression analysis is one method for indirectly obtaining utility values. The mapping regression results (algorithm) can then be applied to other trials and settings where the preference-based measures are missing. The National Institute of Health and Clinical Excellence (NICE) in UK recommends that EQ-5D-3L as one of the most widely used and preferred generic preference-based measure that can generate utility values. Also, EQ-5D-3L can be estimated from another preference-based measure using statistical mapping when it is not available in the relevant study [35]. In FROI analysis, we used mapping from one of the Project outcome measures for depression and anxiety, PHQ-9 to EQ-5D-3L.

In the first stage we estimated FROI ratios for each Project based on the *Depression and Anxiety* domain using the PHQ-9, a widely used tool for assessing the severity of depression [32]. Each of the 9 items on the questionnaire corresponds to one of the DSM-V criteria for depression [36]. PHQ-9 scores were converted to EQ-5D-3L utility values, change in utility values were estimated and adjusted for average annualised changes. Project outcomes as average annualised changes were monetised using QALY threshold value. This process captures the project's overall benefits in monetary terms, comparing them with the investment to estimate value for money.

In the second stage we estimated a single pooled FROI ratio at the overall Program level. There were significant variations across the Projects based on their context, purpose, study design, sample size, targeted outcomes and pre to post intervention time periods. A meta-analysis was conducted using a random effects model including 11 Projects. FROI could not be performed with all the 15 Projects due to factors such as the absence of an appropriate outcome measure to use in the calculation or insufficient sample size.

### 4.2. Estimating FROI Ratio at Project Level

#### 4.2.1. Project characteristics.

The Projects exhibit significant heterogeneity across many categories. The organisations developing and implementing the Project ranged from universities, not for profit to government organisations (e.g., employers). There was a difference in the interventions in terms of the: mechanisms of change, communication channels, and parameters of the exposure to the intervention (i.e., exposure and frequency) [37].

The target audience of the majority of Projects were located at the regional level (in one state or province), or nationally across one country.

#### 4.2.2. Study design and sample size.

Based on data availability, outcome measure suitability, and overall appropriateness, we were able to include only 11 Projects funded through the VFR Grant Program in the analysis. Other Projects used different outcome measures, such as resilience or social support, that could not be translated into HRQol utility values for FROI estimation. Among these 11 Projects there is variation in study design, where some Projects used a single group (cohort) repeated measures design, others used a ‘two-parallel groups’ repeated measures design with the total cohort composed of an intervention group and a control groups (wait list). In both study designs, participants were assessed both at baseline (Time 0: pre-intervention) and at post-intervention (Time 1: post-intervention). For some projects, participants were also assessed at post-post intervention (Time 2: post-post intervention). To facilitate comparison between projects, we have used only two-time points data for this FROI analysis: the baseline (T0: pre-intervention) and one time point in the post-intervention period (either T1: post-intervention or T2 post-post intervention), depending on the appropriateness of the available data for analysis.

Table 1 below presents the variation in study design, sample size and pre to post intervention time among the individual projects.

**Table 1:** Sample size, study-design and pre- to post intervention time for individual projects.

Projects	Study Design	Sample Size (No of participants)	Pre to post intervention time
1	Two-parallel groups’ repeated measures design	For Police and firefighters: 116 in the control and 128 in the intervention group. For Superintendents: 45 in the control and 85 in the intervention group. For Significant others: 2 in the control and 5 in the intervention group	7.7 weeks
2	Single group repeated measures design	112 in the pre- and post- surveys	6 months
3	Single group repeated measures design	183 in the pre- and post- surveys	3 months
4	Two-parallel groups’ repeated measures design	224 in the control group and 144 in the intervention group	3 months
5	Two-parallel groups’ repeated measures design	18 in the control group and 8 in the intervention group	2 months
6	Two-parallel groups’ repeated measures design	110 in the control group and 325 in the intervention group	6 months
7	Single group repeated measures design	49 in the pre- and post- surveys	3 months
8	Single group repeated measures design	28 matched participants in the pre- and post- surveys	6 months
9	Single group repeated measures design	6 matched participants in the pre- and post- surveys	1 month
10	Single group repeated measures design	31 in the pre- and post- surveys	4 months
11	Single group repeated measures design	15 in the pre- and post- surveys	3 months

Note: These studies have not been published yet.

As Table 1 presents, the sample sizes vary across the Projects. In some Projects the sample size is quite small with only 6 matched participants in a single group in the pre- and post- surveys, while in others sample sizes vary involving a maximum of 110 matched participants in the control group and 325 in the intervention group in the pre- and post- surveys.

#### 4.2.3. Estimating the Average Annualized Utility Gain (AAUG).

The perspective of the economic analysis in the VFR evaluation was the funder (not the society or the individual). We aimed to identify the funder’s return on investment (FROI) and compare projects and the grant program. To ensure comparability across interventions of different short durations, we estimated the *Average Annualized Utility Gain (AAUG)*. As in many cases in the VFR Evaluation, pre- and post-intervention periods are determined by real-world implementation science factors rather than randomised controlled trial designs. We first estimated the mean difference in EQ-5D-3L utility values between baseline (T0) and follow-up (T1), then extrapolated this change to a full year to estimate AAUG, assuming the observed improvement at 2-, 3-, 4-, or 6-months post-program remains constant over the year.

Unlike total QALY gain, which reflects overall benefit over a specific period, AAUG expresses the benefit on a per-year basis, enabling fair comparison across interventions with varying follow-up lengths. The process converts utility gains observed during a shorter trial into an annual equivalent by scaling up the observed change. Practically, this involves dividing the utility gain by the fraction of a year represented by the study period.

Following the NICE, 2023 [35] guideline that allows conversion from other scales to EQ-5D-3L utility values where appropriate, we converted PHQ-9 scores, that were commonly available for most of the Projects, to EQ-5D-3L utility values using the algorithm provided in Mukuria et al, 2024 [34]. We estimated the mean difference in EQ-5D-3L utility values between T0 and T1 (for the single group repeated measures design) and difference-in-differences (for the two-parallel groups’ repeated measures design) and extrapolated it to a full year to calculate AAUG.

#### 4.2.4. Estimating AAUG.

The AAUG for the individual Projects were calculated as follows:


$$\begin{array}{cc} & Average\;Annualised\;Utility\;Gain\;(AAUG)\;=\;(Utility\;at\;T\text{1}\;-\;Utility\;at\;T\text{0})\;\times \;\text{1}\text{2}\;months\;(or\;\text{5}\text{2}\;weeks)/\;\\ & the\;time\;gap\;in\;months\;(or\;weeks)\;between\;T\text{1}\;and\;T\text{0} \end{array}$$

#### 4.2.5. Valuing AAUG.

We monetized the AAUG applying the QALY threshold value for Australia expressed in USD, 2019 using relevant health economics literature [38] and adjusted for 2021 using Consumer Price Index for Australia. The adjustment to 2021 USD was required since the funding was allocated in the year 2021.

#### 4.2.6. Estimate FROI Ratio.

The FROI ratio for the individual Project was estimated dividing the quality-of-life value created (monetized value of outcome) by the total investment (monetary and non-monetary costs). This provided a standardized measure of the quality-of-life return on investment for the Project that can be compared with other Projects across the VFR program.


$$\begin{array}{cc} & FROI\;Ratio\;=\;Total\;Value\;Created\;(=\;Monetised\;AAUG\;\times \;Baseline\;implementation\;sample\;size)\;÷\\ & \;Total\;Investment\;(=\;Funding\;Provided\;by\;the\;awarding\;agency/Overall\;Project\;Funding) \end{array}$$

The FROI ratio obtained in this analysis for the individual Projects (11 projects) indicates the overall value generated by a Project compared to the investment made, for example, the value generated for every funder dollar (or total Project dollar) invested. However, in this case the scope of the overall social value generated (the social, environmental, and economic values) by a Project is rather limited to overall quality-of-life impact since we were only able to quantify the *Depression and Anxiety* domain using the Patient Health Questionnaire-9 (PHQ-9), while some interventions in this analysis were not targeted to improve the *Depression and Anxiety* domain.

The FROI ratio quantifies the quality-of-life years gained from investments. Here's how we interpret it: FROI > 1: the Project generates more health value than its cost, indicating a positive return.; FROI = 1: the Project breaks even, generating health value equivalent to the investment; FROI < 1: the Project generates less health value than the investment, suggesting it may not be cost-effective. Some Projects may achieve negative value for FROI, indicating that the Project generating health value less than the resources invested. In other words, the investment is resulting in a net loss of health value. This could mean that the activities are causing more harm than good or that the benefits are not sufficient to justify the costs

The ratio helps stakeholders assess whether the outcomes justify the resources invested. The estimated FROI ratios for the 11 Projects included in this analysis are presented in Table 2 below.

**Table 2:** Estimated FROI ratios for the individual projects.

Projects	Funding from the awarding agency USD	In-kind from the Project USD	Total USD	Time gap pre and post intervention	Baseline sample size	Effect Size (in Utility Scores)	Estimated Benefits in USD	FROI for the awarding agency funding (with 95% CI)	FROI for total Project cost including the in-kind (with 95% CI)
1	318048	164907	482955	7.7 weeks	656	0.025355	5917103.80	18.60 [7.87, 29.34]	12.25 [5.18, 19.32]
2	356349	281474	637823	6 months	232	0.079002	1917741.37	5.38 [4.29, 6.47]	3.0 [2.34, 3.61]
3	357419	110058	467477	3 months	2208	−0.00703	−3246028.25	(-)9.08 [−23.61, 5.44]	(-)6.94 [−18.05, 4.16]
4	187638	34593	222231	3 months	786	0.016347	2688729.75	14.33 [.077, 28.58]	12.09 [.06, 24.13]
5	315697	85321	401018	2 months	56	0.065058	1143602.75	3.62 [.20, 7.04]	2.85 [0.16, 5.54]
6	275,354	152,148	427,502	6 months	500	0.01242	11038.68	0.040 [−3.34, 3.42]	0.026 [−2.15, 2.20]
7	280278	0	280278	3 months	1425	−0.00159	−472678.72	(-)1.69 [−15.00, 11.63]	(-)1.69 [−15.00, 11.63]
8	435534	39790	475324	6 months	36	0.112752	424709.56	0.98 [.38, 1.58]	0.89 [.34, 1.44]
9	308196	0	308196	1 month	29	0.006279	114306.17	0.37 [−3.81, 4.55]	0.37 [−3.81, 4.55]
10	329914	8190	338104	4 months	45	0.072032	508740.34	1.54 [.96, 2.13]	1.50 [.93, 2.07]
11	71123	3000	74123	3 months	26	0.022986	125064.242188	1.76 [−2.25, 5.76]	1.68 [−2.19, 5.53]

Note: Australian Cost-effectiveness threshold per QALY in USD (2019) adjusted for 2021is $52316.00

### 4.3. Estimating FROI Ratio at VFR mental health program level

In order to estimate a single pooled FROI ratio at the overall Program level while we find variations across the individual Projects based on their context, purpose, study design, sample size, targeted outcomes and pre to post intervention time, we conducted a meta-analysis including the 11 individual projects.

**Fig 1:**
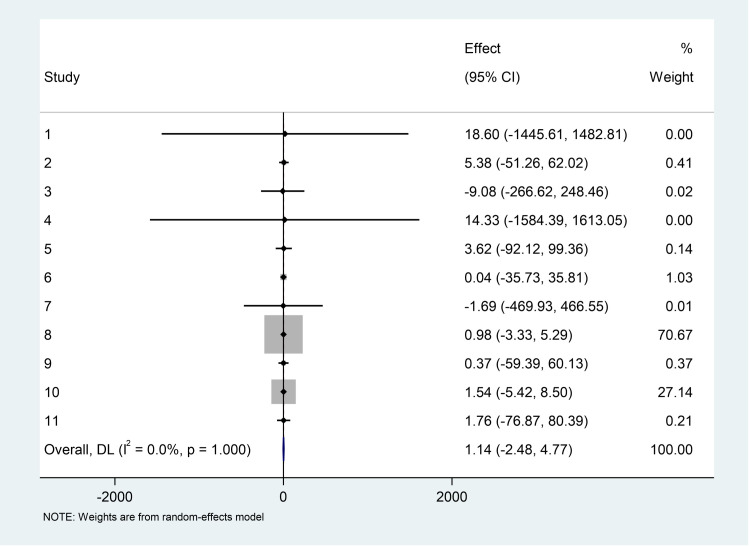
Forest Plot of the Pooled FROI Estimation for VFR Program-Level Funding Provided by the Awarding Agency.

**Fig 2:**
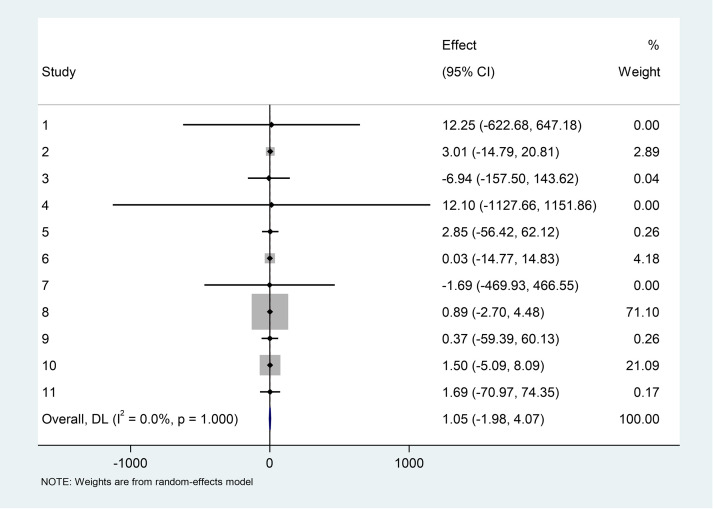
Forest Plot of the Pooled FROI Estimation for VFR Program-Level Funding, Including In-Kind Contributions.

Random effects model using DerSimonian and Laird method was fitted to the data [39]. The inverse of the Freeman–Tukey double arcsine transformation was used to stabilize the variance of each study [40]. Forest plot with the individual study FROI estimates and the 95% confidence interval and the overall pooled FROI estimated at the Program level were presented (Fig 1 and 2). The Z-statistic was used to test the overall effect. Heterogeneity across studies was calculated using the I^2^ statistic [41]. Meta-analysis was performed using Stata 16.1 software (StataCorp, College Station, TX) using the *metan* command, combining results from multiple studies. It's a Stata meta-analysis command useful for pooling estimates from different studies and generating summary statistics, like combined effect size and confidence intervals.

## 5. Results

The information on the individual Projects incorporated in the meta- analysis are presented in Table 2, such as the costs (both funding from the awarding agency and the overall Project funding used independently), the total estimated benefits and the FROI ratios (for the funding agency and the overall Project used independently) with their 95% confidence intervals were incorporated in the meta-analysis. It is evident that FROI values for each dollar invested by the funding agency vary widely, ranging from as high as 18.6:1 to negative in some projects.

Fig 1 and 2 below presents forest plots for Meta-analysis pooling of aggregate data using the random-effects inverse-variance model, respectively for the awarding agency funding only, and for the overall Project funding including the in-kind contributions. As it is evident the pooled FROI at Program level for the funder is 1.14:1 (Fig 1) and for overall funding is 1.05:1 (Fig 2).

Fig 2 below presents forest plot for Meta-analysis pooling of aggregate data using the random-effects inverse-variance model, for the overall Project funding including the in-kind contributions.

This means that for every $1 invested in the VFR Mental Health Program by the funder, there is an overall health value return of $1.14. In other words, the Program generates slightly more value than the resources invested, at this point in time. For example, if $1 million is invested, the Program generates $1.14 million in health-related quality of life value. This suggests that the Program is creating positive value but at a relatively modest level. Likewise, for the overall funding (which included in-kind contribution from the multiple projects, broader investments, or initiatives, every $1 invested results in an overall health return of $1.05. This is a very narrow positive return, meaning the overall initiative is generating just slightly more value than the investment. For example, if $10 million is invested across all programs, it generates $10.5 million in overall health value. In general, VFR Program is performing well, with both the Program and overall funding generating positive returns. However, the returns are close to 1, indicating that the impact, while positive, is relatively modest. Essentially, the programs are nearly breaking even, with only a small net positive effect.

In both these meta-analyses (awarding agency funding only and overall Project funding including the in-kind contributions) obtaining I² (%) = 0.0% means that there is no observed heterogeneity among the studies included in the meta-analysis. This suggests that the effect sizes (e.g., FROI estimates) from the individual studies are consistent with each other, and any variation is likely due to random sampling differences rather than actual differences in effect sizes. Heterogeneity may still exist due to unmeasured factors (e.g., different settings, populations, or measurement techniques), and within-study variation (such as differences in how the intervention is applied or measured across participants).

We performed a sensitivity analysis by conducting the same meta-analysis, first, one project with extreme effect sizes was excluded from each end, followed by the exclusion of two such projects from both ends (for both awarding agency funding only and overall funding including the in-kind contributions from the projects). In both cases, no significant variation in pooled FROI values at the Program level was observed.

## 6. Discussion

Economic evidence is increasingly being used for improving the effectiveness and accountability of programs and policies in the health and social sectors. Despite the availability of international guidelines for health economic analysis, current methods and tools are insufficient for comparing efficient use of resources and value for money across sectors, programs, and countries. This is mainly due to differences in the terminology of service provision and its impact on the analysis of resource utilisation and related costs [42], different approaches to the calculation of health expenditure and financing [43], and the differences in weights and standards of wellbeing/quality of life measures used in cost-utility analysis [29,44] and quality improvement [24]. This largely unexplored research area has a major significance for international funding agencies, interested in improving their funding policy and evaluation of international programs, such as estimating the value for money for their 2021 VFR Mental Health Program [45,46].

Previous health economic analyses in implementation research have concentrated on financial returns, cost-benefit, cost-effectiveness, and cost-utility analyses. Additionally, they have addressed cost minimization, cost-consequence, efficiency, productivity analyses, and Social Return on Investment. To our knowledge, there has not been any prior attempt to analyse the return of investment from the perspective of the funding organisation despite its importance for major public, private and philanthropy organisations in the health and social sectors. Different perspectives, such as societal, public agency, or provider, offer unique insights and implications. For instance, a societal perspective encompasses all costs and benefits to society, including indirect effects like productivity losses, while a public agency perspective focuses on the costs and benefits relevant to public health organizations [27]. From a provider perspective, such as the National Health Services (NHS) in the UK or a single healthcare provider elsewhere, the financial implications include treatment costs (e.g., medication, administration, and monitoring), other health service resource use costs (e.g., GP visits, hospital admissions), and costs associated with managing adverse events caused by treatment. It does not include patients’ costs of obtaining care such as transportation, over the-counter purchases, co-payments or time off work. Whereas, EQ5D-3L utility values are based on the general population’s valuation of health outcomes (obtained through surveys), and not patients own valuations of their health states. Applying this in FROI estimation while focussing on the costs from funder’s perspective also allows for a comprehensive evaluation of the impacts of healthcare interventions, using AAUG for outcome measurement based on general population valuation. The choice of perspective in an economic evaluation depends on context, stakeholder viewpoints, resource availability, and the intended use of the analysis [27]. This study further demonstrates the feasibility of conducting FROI even across a set of highly heterogeneous Projects conducted in 7 different countries and with different targets and samples.

The funder’s perspective for a multi project comparative evaluation is a new field. In estimating the FROI, we adopted a more flexible approach. While not strictly adhering to established economic evaluation methods, this approach remained closely aligned with cost-utility and cost-benefit analysis principles. It facilitated a practical methodology for evaluating real-world implementation research and enabled comparisons between Projects from a funder's perspective.

In general, estimating cost of resource use involves identification, measurement, and valuation of costs. The resultant can then serve as part of economic evaluation. However, there is a scarcity of standardised, generic framework or methodology for resource use and measurement for economic evaluation of programs. The process involves a lot of uncertainty due to the context of the Project and the target audience to whom the results apply. In the case of VFR Grant Program, the target audience varies, for example, veterans, paramedics and fire fighters, their family including their respective stakeholders. Additionally, the Projects differ significantly in understanding expected changes, valuing what matters, and determining material outcomes. Therefore, the FROI analysis is based on real-world and its complex practicalities rather than on standard and fixed methodologies [45]. While the Projects are truly heterogeneous in nature and vary across many categories, in estimating FROI ratios, we aimed to standardise resource utilisation and cost with a focus on the funder’s perspective.

Likewise, there are no internationally established standard guidelines for comparing the Program outcome assessment tools in the form of health utility value [29,44]. A quality-of-life measuring tool called EQ-5D with 5 dimensions and 3 levels of severity is widely used (upgraded to 5 level of severity). This includes generic questionnaires to assess the patients’ health states and estimate utilities. The utilities are used to calculate QALYs, a widely used measure of economic assessment. This helps those conducting the analyses, as well as stakeholders that need to interpret their output as a cost per QALY gained, net monetary benefit or return on investment. The EQ-5D has been used worldwide and directly converted into country-specific single index values (utilities) using country specific value sets. However, only few countries have published country-specific value sets [29]. In analysing international Grant Programs, country-specific standardised value sets are required for comparability. However, none of the VFR Projects collected EQ-5D health state scores or other health/social outcomes measures that can be transferred into utility values, and this was a limiting factor. Consequently, we adopted a novel approach by using a standard scale (PHQ-9) to measure mental health outcomes across the projects. These outcomes were then translated into EQ-5D utility values using statistical mapping and the UK value set, reflecting a standardized measure of how individuals perceive their overall health and its impact on their ability to lead fulfilling lives. We then adopted a simple yet innovative methodology that will support real-world implementation research evaluation and comparisons. This involved estimating the mean difference in quality-of-life utility scores between the pre- and post-intervention periods (in months), as the Projects significantly vary in the length of these periods and adjusting for average annualised gain – AAUG. The concept is well established, as previous peer-reviewed cost-effectiveness studies report “annualized QALY” or similar metrics to standardize QALYs to a yearly basis for comparability. For example, Murphy et al. [47] scaled QALY changes from 12- and 36-week follow-ups to annual equivalents, and subsequent work in addiction research applied the same approach. Likewise, Visco et al. [48] explicitly present *annualized QALY values* derived from trial-based utility measurements to compare outcomes across study arms, demonstrating routine use of this per-year standardisation approach in clinical cost-effectiveness analyses. In addition, Liu et al. [49] provide a formal definition of annualized QALYs within a model-based economic evaluation—calculated as total discounted QALYs divided by the number of years- thereby offering methodological validation of the practice in health-economic modelling. Consistent with these peer-reviewed literature, NICE guideline evidence reviews [50] also annualise utility impacts when incorporating short-term decrements (per-day or per-event) into economic models – typically by converting daily utility losses into an annualised QALY decrement (e.g., per-day QALY loss × 365). This approach is particularly useful for economic evaluations because decision-makers often consider cost per QALY per year or comparable annual health benefit, rather than benefits limited to trial duration.

However, by applying a set of broad, methodologically sound adjustments that are able to capture the characteristics of most Projects included in the FROI analysis, we are able to provide the funder with guidance that is both meaningful and robust. Yet, four Projects were unable to be rated due to insufficient data or no suitable outcome measures for estimating FROI. Therefore, it is recommended funders should enable longitudinal data collection and evaluation with more accurate monitoring of Program effectiveness over time for example enabling data collection at least over three time points – pre, post and post-post implementation timeline. Policy makers should aim to embed economic evaluation requirements into Program design and commissioning, ensuring that new initiatives are evidence informed and evidence generating from the outset. Also, Project implementers should regularly adopt a data-driven decision-making approach, using routinely collected health and administrative data to guide prioritization and service improvement.

Our findings demonstrate a varied range of returns on investment across the projects. The variability in FROI ratios highlights the importance of ongoing assessment and refinement of strategies to maximize the value generated from each Project and to support funder’s decision making on prioritizing funding based on the value for money. There are several other factors that contributed to the FROI estimates. The funding for one Project was for the evaluation component only, thus the FROI calculation only produces a partial estimate without considering the costs of implementing the intervention. So, we need to interpret this estimate with caution. Also, when we considered estimating the FROI incorporating the in-kind and other associated costs from the Projects themselves beyond the awarding agency funding (in-kind costs, funds provided for other sources etc), there was no consistent pattern in which the Project in-kind costs were collected. Thus, the total Project costs used for the second FROI calculation (total cost versus awarding agency funding) has limitations.

Lastly, another innovation in the FROI analysis comes from adopting meta-analysis to estimate a pooled FROI estimate at the overall VFR Program level for the funding agency, which would otherwise not have been feasible. Given the heterogeneity of the Projects and variation in study design and pre- to post intervention periods, it was not practically feasible to aggregate the health outcome of all 15 Projects at the overall VFR Mental Health Program level to estimate an aggregate AAUG for estimating a pooled return on investment ratio.

### 6.1. Limitations

By dividing the mean difference in quality-of-life scores by the length of the pre- to post intervention period (in months) and multiplying by 12, we assumed that the change in health utility values occurs linearly over time. In other words, we assumed that the improvement or decline in quality of life progresses at a constant rate over the length of the pre- to post intervention period and would continue at that rate for a full year. However, the improvement in quality of life might not be linear. For example, the greatest improvement might occur early on after the intervention (e.g., in the first 1–2 months), followed by a plateau, or there may be an increase with improvement over time (e.g., if participants skills to manage their mental health improved over time) or even a decline. If this is the case, this method might overestimate or underestimate the true quality of life gain over time. Nonetheless, for standardizing and comparison where pre- to post data were only available for 2 time points and where the length of the pre- to post intervention period varied widely, this approach allowed us to standardise a common time horizon for all studies (i.e., 1 year), making it easier to compare interventions with different follow-up periods while this method does not account for different patterns of recovery or progression in different interventions. To facilitate the conversion to AAUG, we considered only the pre- to post-improvement in mental health outcomes measured by the PHQ-9 scale, or by concurrent measures of depression which could be converted into PHQ-9 scale score. Other Projects used different outcome measures, such as resilience or social support, and were not necessary targeting depression and anxiety. Therefore, this might explain the lower values recorded in the FROI.

## 7. Conclusion

This study developed a novel and practical framework for estimating the return on investment for international funding agencies, aimed at improving their funding policies and evaluating international programs. The study adopted a flexible approach to capture Projects methodological variations and to standardize resource utilization, valuation, and outcome assessment. It ultimately combined a set of heterogeneous Projects to successfully estimate an overall pooled estimate or return on investment at the Program level. Furthermore, the study highlighted the importance of using novel methods to accommodate heterogeneity, enable standardization and facilitate comparison in real-world implementation research.
