# Supplementary material for: Valuing impact: Estimating return on investment of mental health and wellbeing projects for veterans and first responders
Source: PLoS One. 2026 Aug 5;21(8):e0353179. doi: 10.1371/journal.pone.0353179 (PMC13440868; doi:10.1371/journal.pone.0353179)
Supplement: Supplementary file 1 [file pone.0353179.s001.docx]

Thank you for the opportunity to review your manuscript**: Valuing Impact: Estimating Return on Investment of Mental Health and Wellbeing Projects for Veterans and First Responders.**

In general, I find it well written, with solid explanations and arguments. You are tackling a complex topic, but developing methods to assess impact, value for money, and ROI is very much needed, given the pressure on funding and charity expenditure. I commend your efforts in doing so.

I have provided some insights and comments below to help you improve the manuscript. The main observations I have are about the way you have bounded the problem (a necessary task but not without difficulties), how the application across programs is done (consistently and uniformly, but not without issue) and the way you discuss the utility of the information/your findings.

**Bounding the problem**

You have created an FROI, which you claim measures the return on investment for the funder. The funder’s inputs are easily captured, as well as the inputs of others (in-kind etc). The difficulty arises when you aim to capture outcomes/impacts and determine which of them align with the funder's perspective (which is important to them).

You argue:

Page 3 Line 84 *Evaluating the return on such investments is crucial to ensure the sustainability and scaling of impactful programs, as it helps funders and policymakers understand their economic and social value.*

Page 3 Line 82 *Investing in programs aimed at mental health support and resilience-building for VFRs not only improves individual outcomes but also enhances their capacity to serve society effectively [10].*

Both mention social value; however, you have bounded your analysis to not include social value. Instead, you only focus on individual outcomes, specifically depression and anxiety, extrapolated to QALY.

You state:

Page 6 Line 162 *Movember Foundation, an international philanthropic organisation with a focus on men’s health, conducted an international grant program focused on addressing mental health, wellbeing, and suicide prevention among Veterans and First Responders.*

This gives some indication of the funder’s objectives, and which outcomes may be important to them. If they have funded a program, I would suggest they are interested in the outcomes that program seeks to deliver, including those beyond depression and anxiety. The grant program aims suggest they seek a broader set of outcomes that depression and anxiety. I acknowledge the complexity in harmonising outcome variables here. My point is, that if you are taking the funders perspective you should state how you determined what that perspective is. If you have assumed it, or taken a pragmatic approach based on the available data and variables, you should say so. Or if you undertook activities to understand what (outcome or outcomes) are important to the funder, you should state that (e.g., document reviews/grant guidelines/ funder consultations). If you have formed an understanding and then delimited it, you should say so.

I recommend you add this detail to the manuscript and transparently report your bounding/delimitation process.

**Application across programs**

I commend your efforts to evaluate the entire grant program, given the different programs within it.

You clearly state:

Page 3 Line 102: *challenges are even more pronounced when attempting comparative economic evaluations across a range of projects, given the heterogeneity in project settings, resource use and outcome measurement approaches.*

And explain the outcome measure you selected:

Page 9 Line 234 *FROI analysis, we used mapping from one of the project outcome measures for depression and anxiety, PHQ-9 to EQ-5D-3L*.

This leads me to wonder – how many other project outcomes, related to mental health well being, and suicide prevention, were not included in your analysis? I am particularly concerned given the population of interest (veterans and first responders) who are at risk due to trauma, identity loss, lack of belonging and social isolation, which may contribute to mental health issues and suicide risk, but not through the mechanisms of depression and anxiety.

And later you return to the point about heterogeneity:

Page 20 Line 408 *Other projects used different outcome measures, such as resilience or social support, and were not necessary targeting depression and anxiety. Therefore, this might explain the lower values recorded in the FROI.*

(note the ‘necessary’ should be ‘necessarily’ in the previous sentence)

However, in the analysis, you provide very little detail about the projects. Can you provide more details about the programs/projects' aims? What were they seeking to achieve? Which had depression and anxiety as outcome measures, and which did not?

Although it is ideal to apply an FROI across all programs, the risk is that you have valued the wrong outcome for that program, and in doing so, ‘de-valued’ the program in the funders' eyes (or more broadly) when that program may be delivering greater value against other outcomes. You should articulate this risk.

**Utility of the information/your findings.**

You state:

Page 3 Line 93 *Conducting comparative economic evaluations of implementation strategies is crucial to providing payers, policymakers, and providers necessary insights to determine whether specific strategies represent an efficient use of limited organizational resources.*

However, you do not go on to give any insights as to how your findings are useful for any of these audiences. What recommendations can you make? Where is the so what from your findings? How do you then make recommendations about efficient use of finding dollars, and with what cautions?

I recommend adding text to clearly elucidate the ‘so what’ of your findings.

Some other minor observations that reduce clarity:

Page 8 Line 214 *The standardised mental health outcome is translated into Health-Related Quality of Life (HRQoL) and Quality Adjusted Life Years (QALY).*

At this point in the manuscript, I understand you are seeking to find a measure you can standardise. But the sentences read as if you are already talking about a standardised measure, but have not named it. Consider rewording, given that you introduce the measure you have selected later on.

You state:

Page 7, Line 245 *FROI could not be performed with all the 15 projects due to factors such as the absence of an appropriate outcome measure to use in the calculation or insufficient sample size.*

Which was it? Could you add the number after each factor? E.g., *the absence of an appropriate outcome measure (n=x)*

I wish you well with your revisions.
