# Supplementary material for: Valuing impact: Estimating return on investment of mental health and wellbeing projects for veterans and first responders
Source: PLoS One. 2026 Aug 5;21(8):e0353179. doi: 10.1371/journal.pone.0353179 (PMC13440868; doi:10.1371/journal.pone.0353179)
Supplement: Supplementary file 2 [file pone.0353179.s003.docx]

# Statement Addressing the Reviewer’s Comment:

We thank the Editor and Reviewers for their thoughtful feedback and the opportunity to revise our manuscript. We believe that addressing these comments has substantially strengthened the revised submission. Please note that all page and line numbers referenced in this document correspond to the *Revised manuscript with track changes enabled*.

We would also like to note that we took the opportunity to refine the terminology and to clarify in detail in the revised manuscript that our analyses used **utility values (average annualised adjustments)** rather than **changes in QALYs**, in order to enable valid comparisons across heterogeneous projects. To further enhance clarity and avoid potential misinterpretation, we have replaced the term *“mean annualised QALY gain”* with **Average Annualised Utility Gain (AAUG)**, thereby clearly distinguishing this measure from absolute QALY gains. These changes are reflected all through the document on ***Methods*** and ***Discussion*** section

.

- Reviewer’s Comment (1):

Problem Bounding and Funder's Perspective: While you claim to measure ROI from the funder's perspective (FROI), the reviewer notes a critical disconnect. Your analysis focuses solely on depression and anxiety outcomes (converted to QALYs), yet the Movember Foundation's grant program explicitly targets "mental health, wellbeing, and suicide prevention." You must:

- Comment (1a): Clearly explain how you determined what outcomes matter to the funder

***Response:*** We thank the reviewer for this comment, and we have now added a section on page 4 titled ***Study Context*** and paragraphs on page 7-8 line 206-216, page 9-10 line 256-288 addressing the reviewer’s comment in the revised manuscript. In addressing the reviewer’s comments in this response document, we also wish to contextualise the analyses with the following explanations.

This manuscript forms part of a broader, multi‑layered evaluation of the Movember and Distinguished Gentleman’s Ride–funded Veteran and First Responders (VFR) Mental Health Program. As already stated in the manuscript, the overall Program supported 15 organisations across seven countries to deliver early‑intervention Projects aimed at improving the mental health and well‑being of VFRs and their families or significant others. The evaluation protocol, including the novel external evaluation and impact analysis methods, has already been published elsewhere (Lukersmith et al, 2025a).

The reviewer is correct in noting that the Grant Program is centred on supporting mental health, wellbeing, and suicide prevention among Veterans and First Responders. However, it is important to clarify that the scope of this evaluation necessarily extends beyond documenting mental health outcomes alone. In line with Movember’s objectives and its commitment to impact accountability, the evaluation examines which Projects are effective, sustainable, scalable, and represent value for money. It also assesses overall Program performance and the return on investment (ROI) for the Grant Program as a whole (Lukersmith et al., 2025a).

This broader evaluative lens is essential. It allows us to understand not only **whether** improvements occurred, but also **how**, **why**, and under what **conditions** these improvements can be maintained, replicated, or expanded in a cost‑effective way. Estimating the funder’s ROI represents one component of this larger evaluative framework, and Movember was specifically interested in understanding the monetary value of its investment relative to measurable participant outcomes.

To conduct this analysis rigorously, it was necessary to focus on outcome measures that were consistently collected across the majority of funded Projects- particularly PHQ‑9 and GAD‑7 scores- because these outcome measures can be reliably mapped to utility values and subsequently assigned with the QALY threshold value. Without this harmonisation, it would not be possible to derive a credible or comparable ROI for the Program.

We therefore hope this addresses the reviewer’s concern regarding a “critical disconnect” between the FROI analysis and the funder’s perspective. The use of depression and anxiety outcomes (converted to utility values) reflects not a narrowing of Program objectives, but the methodological requirements for producing a robust, comparable, and funder‑relevant assessment of ROI within a diverse multi‑project grant portfolio.

- Comment (1b): Transparently report your delimitation process

***Response:*** We thank the reviewer for this comment, and as mentioned in response to earlier Comment (1a) we have now added paragraphs on page 9-10 line 256-288 addressing the reviewer’s comment in the revised manuscript explaining the delimitation process.

The evaluation encountered considerable complexity due to the heterogeneity of the 15 projects, each operating within different organisational, cultural, and system environments and commencing at staggered times during 2022-2023. Additional challenges included the absence of common outcome measures, differing ethics and governance requirements, staffing delays, and coordination demands across multiple international time zones. These limitations could have compromised comparability and rigour if not addressed systematically.

In response, the evaluation team developed a comprehensive and methodologically robust roadmap to ensure integrity and consistency across the Program. This roadmap combined innovative analytical approaches, a co‑design methodology, and sustained engagement with Movember and all Project teams.

This process resulted in the identification of five consistent mental health and well‑being indicators - depression and anxiety, trauma, resilience, support, and quality of life, aligned with Movember’s MO5 behaviour objectives. The outcome measures that were considered concurrent or convergent by the expert panel and thereby could be used as a proxy in the multi-layered statistical analysis, efficiency analysis and Social Return on Analysis evaluations. These indicators provided the foundation for synthesising findings across highly diverse Projects and contexts.

The following activities were performed during this co-design and preparation period:

- Establish a data sharing agreement with each of the 15 organisations and confirm each project has ethical approval and consent forms which includes sharing de-identified data with UC.
- Confirm the logic of change (inputs, throughputs, and outputs), target audience/s and sample size for each of the Projects.
- Gather information relevant to the evaluation e.g. intervention mechanisms, time frames, recruitment period.
- Establish domains of inputs, throughputs and outputs across all Projects and the relevant parameters of some domains (e.g. frequency of best practice range) with a panel of Project’s representatives and Movember.
- Identify the set of outcome measures adopted by the different projects in each indicator, acknowledge the common outcome measure for each indicator for evaluation, using a novel scientific approach of calibration with an expert panel of clinicians and researchers to determine the level of concurrence and convergence on the properties of each outcome measure for a specific indicator.
- Affirm time points that the Projects will be collecting data, the minimum participant numbers for the various analysis methodology (e.g. statistical analysis, SROI)
- Collaborate with the Projects to support their data collection, pro-vide support or advice as needed, develop systems for secure data transfer within the seven country jurisdictions.

Despite the significant variability across countries, project designs, and implementation realities, this evaluation framework enabled the generation of meaningful, comparable, and policy‑relevant insights. The co‑designed, collaborative approach ensured that the evaluation not only met scientific standards but also contributed to collective organisational learning and practical guidance for future investments in VFR mental health.

- Comment (1c): Acknowledge if you took a pragmatic approach based on data availability

***Response:*** We thank the reviewer for this comment. We indeed adopted an ecosystem‑based, real‑world pragmatic approach that recognises context and addresses the complexities inherent in evaluating a multi‑step, mixed‑methods, and highly complex evaluation process. We have addressed this point in our responses to the Reviewer’s earlier comments and have incorporated corresponding text into the revised manuscript on pages 7-8 and 9-10 to clarify this overarching approach.

- Comment (1d): Justify why broader social value was excluded despite being mentioned in your rationale

***Response:*** We thank the reviewer for this comment and appreciate the concern regarding the inability to estimate an SROI within this evaluation. As explained on pages 6-7 (lines 177-185), SROI relies heavily on subjective assumptions, stakeholder input, and the use of proxy monetary values. While these features can be useful for capturing rich, context‑specific impacts at an individual project level, they also introduce considerable variability. The absence of a standardised framework for valuing health outcomes and measuring resource use increases the risk of project‑specific bias. Moreover, monetising non‑market outcomes can oversimplify complex social impacts, making it difficult to generate comparable results across multiple projects—an essential requirement for evaluating the Movember VFR Program as a whole.

- Reviewer’s Comment (2):

Cross-Program Application and Risk of Misvaluation: The reviewer raises serious concerns about applying a uniform outcome measure across heterogeneous programs. You must:

- Comment (2a): Provide detailed descriptions of each program's specific aims and intended outcomes
- Comment (2b): Clearly identify which programs actually targeted depression/anxiety versus other outcomes

***Response:*** We thank the reviewer for this important comment. We agree that addressing it will strengthen the revised manuscript. As noted in our earlier response, this manuscript is part of a broader, multi‑layered evaluation of the Movember and Distinguished Gentleman’s Ride–funded Veteran and First Responders (VFR) Mental Health Program.

The evaluation team identified five domains to assess overall Project and Program performance and enable comparison across heterogeneous initiatives. Funder Return on Investment is one of these domains; the others are Complexity, Implementation Processes, Outcomes, and Efficiency.

We appreciate the reviewer’s concerns regarding the use of uniform outcome measures across diverse programs. However, the identification of domains, indicators, and outcome measures involved multiple stages of innovative analytical work, a rigorous co‑design process, and sustained engagement with Movember and all Project teams. The step‑by‑step protocol and multi‑step comparative evaluation have been published elsewhere (Lukersmith et al., 2025a, 2025b).

To address the reviewer’s comment, we first note that the initial and most relevant step in our process was the identification of evaluation indicators.

The domains were evaluated using a co‑designed indicator set based on a Logic of Change framework (inputs, throughputs, and outputs, including participant outcomes). A multi‑step process was used to develop indicators for the Projects and Program evaluations and to create a common set across all 15 Projects. Initial indicators were drafted by the evaluation team (authors) using the Logic of Change model.

An external peer‑review process was then undertaken to refine and finalise the projects targeted outcome indicators. The Indicators Expert Panel (n=11), comprising evaluation experts, Movember representatives, team members, and external advisors (such as a statistician, evaluation specialist, and mental health expert), began its work in 2021. The panel met twice, supported by follow‑up email consultations for members unable to attend. Through this consensus process, the initial indicator set was revised and reduced to its final form. The five consistent mental health and well‑being indicators include depression and anxiety, trauma, resilience, support, and quality of life.

The second relevant step in this process was identifying and acknowledging the heterogeneity in project participant outcome measures.

Project heterogeneity (e.g., target groups, intervention types) led to wide variation in outcome measures. Although Movember and the evaluation team encouraged two common outcome measures (GAD-7 and PHQ-9 in the space of depression and anxiety that can be treated and translated into overall health related quality of life (HQoL)) to enable cross‑Project comparison, this was not feasible due to differing project aims, concerns about survey burden and ethics, and timing constraints. Consequently, the projects used 120 different outcome measures.

To address this, the evaluation team applied a calibration approach using clinical experts to assess convergence between measures. The process identified six commonly measured indicators: depression, anxiety, trauma‑related stress, resilience, support, and quality of life. Across these, Projects used 39 different outcome measures, and a “common comparator” was selected for each indicator based on a similar External Expert panel peer review process described earlier.

The evaluation team reviewed all outcome measures for constructs, psychometrics, and validity, comparing each with the common comparator (e.g., Patient Health Questionnaire for Depression symptoms (PHQ-2) vs. Center for Epidemiologic Studies Depression Scale (CESD‑R), Beck Depression Inventory II for Depression symptom (BDI_II), Patient Health Questionnaire for Depression symptoms (PHQ‑4)). The expert panel (n=14) assessed concurrence and convergence using nominal group methods over three meetings (Aug-Sept 2023). This enabled evaluation team to compare outcomes across more projects, though some indicators had data from only up to eight projects.

- Comment (2c): Explicitly discuss the risk of undervaluing programs by measuring inappropriate outcomes

***Response:*** In addressing the reviewer’s comment, we emphasise that although FROI was one domain within the broader evaluation, the authors examined all five outcome indicators targeted across the projects—depression and anxiety, trauma‑related stress, resilience, support, and quality of life. Crucially, several of these indicators (e.g., trauma‑related stress, resilience, and support) could **not** be mapped to utility values or converted into Health Related Quality of Life utility values (HRQoL), which are required to calculate a return on investment (ROI) from the funder’s perspective.

For this reason, we selected the PHQ‑9 and GAD‑7 not only because they were identified as the Common Comparators across the multiple OMs within the depression and anxiety domains, but also because they are widely used, condition‑specific symptom measures that can be translated to HRQol utilities for economic evaluation (Mukuria et al, 2024), ensuring that programs are not undervalued by relying on only comparable or inappropriate outcome measures.

- Comment (2d): Consider whether all programs should be included in this analysis framework

***Response:*** As stated in the manuscript and we have now explicitly added a sentence to the *4.22 Study Design and Sample Size* sub-section (p. 12, lines 335-339), that was earlier in the limitation section, FROI analyses could not be conducted for all 15 eligible projects due to factors such as the absence of a suitable outcome measure or insufficient sample size. This limitation reflects feasibility constraints rather than an undervaluation of these programs. Moreover, although these projects could not be included in the FROI calculation, they were fully evaluated across the other performance domains in the overall evaluation framework.

The use of PHQ‑9 and GAD‑7 in the FROI analysis was methodologically justified, consistent with best practice, and necessary to ensure valid and comparable economic estimates, without disadvantaging programs for which economic valuation was not feasible.

- Reviewer’ Comment (3):

Utility and Actionable Insights: Your manuscript currently lacks clear practical implications. You must:

- Articulate specific recommendations for funders, policymakers, and providers
- Explain how your findings can guide efficient resource allocation
- Include appropriate caveats and limitations when making recommendations
- Add a clear "so what" discussion of your results

***Response:*** We thank the reviewer for this insightful comment. In addressing this reviewer’s comment, we have now added two paragraphs on page 24-25 in the discussion section in the revised manuscript.

Reviewer’s Minor Revisions:

- Reviewer’s Comment (4):

Clarify the wording around "standardised mental health outcome" (p.8, line 214)

***Response:*** This has now been addressed in the revised manuscript on page 9-11. Sub-section 4.13, *Outcome Measurement*, has been thoroughly revised, with terminology edited and clarified in detail in the revised manuscript.

- Reviewer Comment (5):

Specify the breakdown of why FROI couldn't be performed for certain projects (p.7, line 245)

***Response:*** As stated in the manuscript (p. 12, lines 335–339), FROI analyses could not be conducted for all 15 eligible projects due to factors such as the absence of a suitable outcome measure that could enable comparison across projects or insufficient sample size. This limitation reflects feasibility constraints. Moreover, although these projects could not be included in the FROI calculation, they were fully evaluated across the other performance domains in the overall evaluation framework.

- Reviewer Comment (6):

Correct "necessary" to "necessarily" (p.20, line 408)

***Response:*** This has now been edited in the revised manuscript.

- Reviewer’s Comment (7):

The reviewer's feedback indicates that while your methodological approach has merit, the current framing and application may inadvertently devalue important programs that address veteran and first responder wellbeing through mechanisms beyond depression and anxiety. This fundamental issue must be thoroughly addressed in your revision.

***Response:*** We that the editor for this overall comment and we believe that we have now addressed these concerns in the revised manuscript. For details, please refer to the newly added texts on page 7-8 line 206-216, page 9-10 line 256-288 and response to Reviewer’s comment 1 and 2 above.

References:

Lukersmith, S., et al. (2025a), *An ecosystem approach to the evaluation and impact analysis of heterogeneous preventive and/or early interventions projects for veterans and first responders in seven countries.* Comprehensive Psychiatry, 2025. **138**: p. 152578.

Lukersmith, S., et al. (2025b), *A comparative evaluation of 23 projects on mental health and wellbeing for veterans and first responders.* Comprehensive Psychiatry, 2025. **141**: p. 152599.
