# Supplementary material for: Valuing impact: Estimating return on investment of mental health and wellbeing projects for veterans and first responders
Source: PLoS One. 2026 Aug 5;21(8):e0353179. doi: 10.1371/journal.pone.0353179 (PMC13440868; doi:10.1371/journal.pone.0353179)
Supplement: Supplementary file 3 [file pone.0353179.s004.docx]

# Response to Reviewer#1 Comments:

We thank the Reviewer for their thoughtful feedback and the editor for providing the opportunity to revise our manuscript. We believe that addressing these comments has significantly enhanced the presentation of the revised manuscript. Please note that all page and line numbers referenced in this document correspond to the *Revised manuscript with track changes enabled*.

- Reviewer #1: Thank you for addressing the reviewer comments thoughtfully. They have addressed my comments substantively.
  Author Response: We thank the reviewer for this encouraging comment.
- Reviewer #1: I found some minor issues that you should address before finalising the manuscript. They are:

Page 2 (abstract), line 77: given you list several reasons, 'contribute' should be plural

Author Response: This is now edited in the revised draft.

- Page 6 line 165: 'monetise' should be monetises (to match 'converts')
  Author Response: We thank the reviewer for identifying this typographical error. It has now been corrected in the revised manuscript on page#6 line#163.
- Page 7, line 185: suggest 'analysis of the 2021 Movember VFR...' which makes it clear that is the funding/granting date, rather than the date of your analysis.
  Author Response: We thank the reviewer for identifying this discrepancy in dates. This sentence has now been edited in the revised manuscript. The revised text on page#6 line#183-184 now reads as “we conduct an economic analysis of the ROI for the funder as part of a broader impact analysis of the 2021 Movember VFR Program with final data collected from each project by March 2024”.
- Page 8 line 217: teams with the full protocol (add 'the)
  Author Response: We thank the reviewer for identifying this typographical error. It has now been corrected in the revised manuscript on page#7 line#198.
- Page 9 line 260: are all 108 outcomes participant outcomes? or were there some program outcomes here? There can be program outcomes that are not participant outcomes - so please check.
  Author Response: This text has now been edited in the revised manuscript on page#8 line#235. Just to confirm, yes, there were120 patient reported outcome measures (PROMs)used across 15 projects for different mental health outcomes.
- Page 10 line 268: and are published elsewhere (add 'are')
  Author Response: We thank the reviewer for identifying this typographical error. It has now been corrected in the revised manuscript on page#9 line#246.
- Page 11 line 295. Sentence starting with 'However...' does not make sense. Please check and clarify.
  Author Response: We thank the reviewer for this comment. It has now been edited in the revised manuscript on page#10 line#270.
- Page 10 line 315: 'change is utility' should be 'change in utility', and there is 'ad' at the end of the sentence before the struck out text
  Author Response: We thank the reviewer for identifying this typographical error. It has now been edited in the revised manuscript on page#10 line#290.
- Page 12 line 337: should clarify were only able to include 11 projects 'in the analysis'
  Author Response: We thank the reviewer for identifying this missing phrase. It has now been edited in the revised manuscript on page#11 line#311.
- Page 16 line 421: substitute 'get' with achieve
  Author Response: We thank the reviewer for this comment. It has now been edited in the revised manuscript on page#15 line#379.
- Page 22 line 314: how many projects excluded in the first sensitivity approach? you note 2 projects in the second approach.
  Author Response: We thank the reviewer for this comment. It has now been edited in the revised manuscript on page#20 line#311-313.
- Page 25 line 410: sentence starting 'Therefore, we encourage..' does not make sense. Please check and clarify.

Author Response: We thank the reviewer for this comment. This text has now been edited in the revised manuscript on page#23 line#404-405.
