# Supplementary material for: Valuing impact: Estimating return on investment of mental health and wellbeing projects for veterans and first responders
Source: PLoS One. 2026 Aug 5;21(8):e0353179. doi: 10.1371/journal.pone.0353179 (PMC13440868; doi:10.1371/journal.pone.0353179)
Supplement: Peer review history

# Peer review: Decision Letter 0

**DOI:** 10.1371/journal.pone.0353179.r001

**Authors:** Sompop Bencharit

26 Nov 2025

PONE-D-25-50425

Valuing Impact: Estimating Return on Investment of Mental Health and Wellbeing Projects for Veterans and First Responders

PLOS ONE

Dear Dr. Mohanty,

Thank you for submitting your manuscript to PLOS ONE. After careful consideration, we feel that it has merit but does not fully meet PLOS ONE’s publication criteria as it currently stands. Therefore, we invite you to submit a revised version of the manuscript that addresses the points raised during the review process.

Please address the following major and minor issues:

**Major issues:**

1. **Problem Bounding and Funder's Perspective:**

While you claim to measure ROI from the funder's perspective (FROI), the reviewer notes a critical disconnect. Your analysis focuses solely on depression and anxiety outcomes (converted to QALYs), yet the Movember Foundation's grant program explicitly targets "mental health, wellbeing, and suicide prevention." You must:

- Clearly explain how you determined what outcomes matter to the funder
- Transparently report your delimitation process
- Acknowledge if you took a pragmatic approach based on data availability
- Justify why broader social value was excluded despite being mentioned in your rationale

2. Cross-Program Application and Risk of Misvaluation:

The reviewer raises serious concerns about applying a uniform outcome measure across heterogeneous programs. You must:

- Provide detailed descriptions of each program's specific aims and intended outcomes
- Clearly identify which programs actually targeted depression/anxiety versus other outcomes
- Explicitly discuss the risk of undervaluing programs by measuring inappropriate outcomes
- Consider whether all programs should be included in this analysis framework

3. Utility and Actionable Insights: Your manuscript currently lacks clear practical implications. You must:

- Articulate specific recommendations for funders, policymakers, and providers
- Explain how your findings can guide efficient resource allocation
- Include appropriate caveats and limitations when making recommendations
- Add a clear "so what" discussion of your results

Minor issues:

- Clarify the wording around "standardised mental health outcome" (p.8, line 214)
- Specify the breakdown of why FROI couldn't be performed for certain projects (p.7, line 245)
- Correct "necessary" to "necessarily" (p.20, line 408)

Please submit your revised manuscript by Jan 07 2026 11:59PM. If you will need more time than this to complete your revisions, please reply to this message or contact the journal office at plosone@plos.org. When you're ready to submit your revision, log on to https://www.editorialmanager.com/pone/ and select the 'Submissions Needing Revision' folder to locate your manuscript file.

Please include the following items when submitting your revised manuscript:

- A rebuttal letter that responds to each point raised by the academic editor and reviewer(s). You should upload this letter as a separate file labeled 'Response to Reviewers'.
- A marked-up copy of your manuscript that highlights changes made to the original version. You should upload this as a separate file labeled 'Revised Manuscript with Track Changes'.
- An unmarked version of your revised paper without tracked changes. You should upload this as a separate file labeled 'Manuscript'.

If you would like to make changes to your financial disclosure, please include your updated statement in your cover letter. Guidelines for resubmitting your figure files are available below the reviewer comments at the end of this letter.

If applicable, we recommend that you deposit your laboratory protocols in protocols.io to enhance the reproducibility of your results. Protocols.io assigns your protocol its own identifier (DOI) so that it can be cited independently in the future. For instructions see: https://journals.plos.org/plosone/s/submission-guidelines#loc-laboratory-protocols. Additionally, PLOS ONE offers an option for publishing peer-reviewed Lab Protocol articles, which describe protocols hosted on protocols.io. Read more information on sharing protocols at https://plos.org/protocols?utm_medium=editorial-email&utm_source=authorletters&utm_campaign=protocols.

We look forward to receiving your revised manuscript.

Kind regards,

Sompop Bencharit, DDS, MS, PhD, FACP

Academic Editor

PLOS ONE

Journal Requirements:

When submitting your revision, we need you to address these additional requirements.

1. Please ensure that your manuscript meets PLOS ONE's style requirements, including those for file naming. The PLOS ONE style templates can be found at

https://journals.plos.org/plosone/s/file?id=wjVg/PLOSOne_formatting_sample_main_body.pdf and

https://journals.plos.org/plosone/s/file?id=ba62/PLOSOne_formatting_sample_title_authors_affiliations.pdf

2. Thank you for stating the following in the Acknowledgments Section of your manuscript:

“This work was supported by the Movember Foundation and The Distinguished Gentlemen’s Ride.”

We note that you have provided funding information that is not currently declared in your Funding Statement. However, funding information should not appear in the Acknowledgments section or other areas of your manuscript. We will only publish funding information present in the Funding Statement section of the online submission form.

Please remove any funding-related text from the manuscript and let us know how you would like to update your Funding Statement. Currently, your Funding Statement reads as follows:

“This work was supported by the Movember Foundation and The Distinguished Gentlemen’s Ride.”

Please include your amended statements within your cover letter; we will change the online submission form on your behalf.

3. Thank you for stating the following financial disclosure:

“This work was supported by the Movember Foundation and The Distinguished Gentlemen’s Ride.”

Please state what role the funders took in the study.  If the funders had no role, please state: "The funders had no role in study design, data collection and analysis, decision to publish, or preparation of the manuscript."

If this statement is not correct you must amend it as needed.

Please include this amended Role of Funder statement in your cover letter; we will change the online submission form on your behalf.

4. We note that you have indicated that there are restrictions to data sharing for this study. PLOS only allows data to be available upon request if there are legal or ethical restrictions on sharing data publicly. For more information on unacceptable data access restrictions, please see http://journals.plos.org/plosone/s/data-availability#loc-unacceptable-data-access-restrictions.

Before we proceed with your manuscript, please address the following prompts:

a) If there are ethical or legal restrictions on sharing a de-identified data set, please explain them in detail (e.g., data contain potentially identifying or sensitive patient information, data are owned by a third-party organization, etc.) and who has imposed them (e.g., a Research Ethics Committee or Institutional Review Board, etc.). Please also provide contact information for a data access committee, ethics committee, or other institutional body to which data requests may be sent.

b) If there are no restrictions, please upload the minimal anonymized data set necessary to replicate your study findings to a stable, public repository and provide us with the relevant URLs, DOIs, or accession numbers. For a list of recommended repositories, please see

https://journals.plos.org/plosone/s/recommended-repositories. You also have the option of uploading the data as Supporting Information files, but we would recommend depositing data directly to a data repository if possible.

We will update your Data Availability statement on your behalf to reflect the information you provide.

5. Your ethics statement should only appear in the Methods section of your manuscript. If your ethics statement is written in any section besides the Methods, please delete it from any other section.

6. If the reviewer comments include a recommendation to cite specific previously published works, please review and evaluate these publications to determine whether they are relevant and should be cited. There is no requirement to cite these works unless the editor has indicated otherwise.

Additional Editor Comments:

Thank you for submitting your manuscript "Valuing Impact: Estimating Return on Investment of Mental Health and Wellbeing Projects for Veterans and First Responders" to our journal.

After careful review, I am pleased to inform you that your manuscript has potential for publication, but requires major revisions before it can be accepted. The reviewer commends your work on this complex and important topic, noting that developing methods to assess impact and ROI is crucial given current funding pressures. However, several significant issues need to be addressed.

Major Issues Requiring Revision:

Problem Bounding and Funder's Perspective: While you claim to measure ROI from the funder's perspective (FROI), the reviewer notes a critical disconnect. Your analysis focuses solely on depression and anxiety outcomes (converted to QALYs), yet the Movember Foundation's grant program explicitly targets "mental health, wellbeing, and suicide prevention." You must:

Clearly explain how you determined what outcomes matter to the funder

Transparently report your delimitation process

Acknowledge if you took a pragmatic approach based on data availability

Justify why broader social value was excluded despite being mentioned in your rationale

Cross-Program Application and Risk of Misvaluation: The reviewer raises serious concerns about applying a uniform outcome measure across heterogeneous programs. You must:

Provide detailed descriptions of each program's specific aims and intended outcomes

Clearly identify which programs actually targeted depression/anxiety versus other outcomes

Explicitly discuss the risk of undervaluing programs by measuring inappropriate outcomes

Consider whether all programs should be included in this analysis framework

Utility and Actionable Insights: Your manuscript currently lacks clear practical implications. You must:

Articulate specific recommendations for funders, policymakers, and providers

Explain how your findings can guide efficient resource allocation

Include appropriate caveats and limitations when making recommendations

Add a clear "so what" discussion of your results

Minor Revisions:

Clarify the wording around "standardised mental health outcome" (p.8, line 214)

Specify the breakdown of why FROI couldn't be performed for certain projects (p.7, line 245)

Correct "necessary" to "necessarily" (p.20, line 408)

The reviewer's feedback indicates that while your methodological approach has merit, the current framing and application may inadvertently devalue important programs that address veteran and first responder wellbeing through mechanisms beyond depression and anxiety. This fundamental issue must be thoroughly addressed in your revision.

[Note: HTML markup is below. Please do not edit.]

Reviewers' comments:

Reviewer's Responses to Questions

**Comments to the Author**

1. Is the manuscript technically sound, and do the data support the conclusions?

The manuscript must describe a technically sound piece of scientific research with data that supports the conclusions. Experiments must have been conducted rigorously, with appropriate controls, replication, and sample sizes. The conclusions must be drawn appropriately based on the data presented.

Reviewer #1: Yes

**********

2. Has the statistical analysis been performed appropriately and rigorously?

Reviewer #1: Yes

**********

3. Have the authors made all data underlying the findings in their manuscript fully available?

The PLOS Data policy requires authors to make all data underlying the findings described in their manuscript fully available without restriction, with rare exception (please refer to the Data Availability Statement in the manuscript PDF file). The data should be provided as part of the manuscript or its supporting information, or deposited to a public repository. For example, in addition to summary statistics, the data points behind means, medians and variance measures should be available. If there are restrictions on publicly sharing data—e.g. participant privacy or use of data from a third party—those must be specified.

Reviewer #1: No

**********

4. Is the manuscript presented in an intelligible fashion and written in standard English?

PLOS ONE does not copyedit accepted manuscripts, so the language in submitted articles must be clear, correct, and unambiguous. Any typographical or grammatical errors should be corrected at revision, so please note any specific errors here.

Reviewer #1: Yes

**********

5. Review Comments to the Author

Please use the space provided to explain your answers to the questions above. You may also include additional comments for the author, including concerns about dual publication, research ethics, or publication ethics. (Please upload your review as an attachment if it exceeds 20,000 characters)

Reviewer #1: Thank you for the opportunity to review your manuscript: Valuing Impact: Estimating Return on Investment of Mental Health and Wellbeing Projects for Veterans and First Responders. Please see attached document for my comments.

**********

6. PLOS authors have the option to publish the peer review history of their article (what does this mean?). If published, this will include your full peer review and any attached files.

If you choose “no”, your identity will remain anonymous but your review may still be made public.

**Do you want your identity to be public for this peer review?**  For information about this choice, including consent withdrawal, please see our Privacy Policy.

Reviewer #1: No

**********

[NOTE: If reviewer comments were submitted as an attachment file, they will be attached to this email and accessible via the submission site. Please log into your account, locate the manuscript record, and check for the action link "View Attachments". If this link does not appear, there are no attachment files.]

To ensure your figures meet our technical requirements, please review our figure guidelines: https://journals.plos.org/plosone/s/figures

You may also use PLOS’s free figure tool, NAAS, to help you prepare publication quality figures: https://journals.plos.org/plosone/s/figures#loc-tools-for-figure-preparation.

NAAS will assess whether your figures meet our technical requirements by comparing each figure against our figure specifications.

Attachment Submitted filename: PLOS review.docx

# Author comment: Author response to Decision Letter 1

**DOI:** 10.1371/journal.pone.0353179.r002

26 Mar 2026

Statement Addressing the Reviewer’s Comment:

We thank the Editor and Reviewers for their thoughtful feedback and the opportunity to revise our manuscript. We believe that addressing these comments has substantially strengthened the revised submission. Please note that all page and line numbers referenced in this document correspond to the Revised manuscript with track changes enabled.

We would also like to note that we took the opportunity to refine the terminology and to clarify in detail in the revised manuscript that our analyses used utility values (average annualised adjustments) rather than changes in QALYs, in order to enable valid comparisons across heterogeneous projects. To further enhance clarity and avoid potential misinterpretation, we have replaced the term “mean annualised QALY gain” with Average Annualised Utility Gain (AAUG), thereby clearly distinguishing this measure from absolute QALY gains. These changes are reflected all through the document on Methods and Discussion section.

� Reviewer’s Comment (1):

Problem Bounding and Funder's Perspective: While you claim to measure ROI from the funder's perspective (FROI), the reviewer notes a critical disconnect. Your analysis focuses solely on depression and anxiety outcomes (converted to QALYs), yet the Movember Foundation's grant program explicitly targets "mental health, wellbeing, and suicide prevention." You must:

- Comment (1a): Clearly explain how you determined what outcomes matter to the funder

Response: We thank the reviewer for this comment, and we have now added a section on page 4 titled Study Context and paragraphs on page 7-8 line 206-216, page 9-10 line 256-288 addressing the reviewer’s comment in the revised manuscript. In addressing the reviewer’s comments in this response document, we also wish to contextualise the analyses with the following explanations.

This manuscript forms part of a broader, multi layered evaluation of the Movember and Distinguished Gentleman’s Ride–funded Veteran and First Responders (VFR) Mental Health Program. As already stated in the manuscript, the overall Program supported 15 organisations across seven countries to deliver early intervention Projects aimed at improving the mental health and well being of VFRs and their families or significant others. The evaluation protocol, including the novel external evaluation and impact analysis methods, has already been published elsewhere (Lukersmith et al, 2025a).

The reviewer is correct in noting that the Grant Program is centred on supporting mental health, wellbeing, and suicide prevention among Veterans and First Responders. However, it is important to clarify that the scope of this evaluation necessarily extends beyond documenting mental health outcomes alone. In line with Movember’s objectives and its commitment to impact accountability, the evaluation examines which Projects are effective, sustainable, scalable, and represent value for money. It also assesses overall Program performance and the return on investment (ROI) for the Grant Program as a whole (Lukersmith et al., 2025a).

This broader evaluative lens is essential. It allows us to understand not only whether improvements occurred, but also how, why, and under what conditions these improvements can be maintained, replicated, or expanded in a cost effective way. Estimating the funder’s ROI represents one component of this larger evaluative framework, and Movember was specifically interested in understanding the monetary value of its investment relative to measurable participant outcomes.

To conduct this analysis rigorously, it was necessary to focus on outcome measures that were consistently collected across the majority of funded Projects- particularly PHQ 9 and GAD 7 scores- because these outcome measures can be reliably mapped to utility values and subsequently assigned with the QALY threshold value. Without this harmonisation, it would not be possible to derive a credible or comparable ROI for the Program.

We therefore hope this addresses the reviewer’s concern regarding a “critical disconnect” between the FROI analysis and the funder’s perspective. The use of depression and anxiety outcomes (converted to utility values) reflects not a narrowing of Program objectives, but the methodological requirements for producing a robust, comparable, and funder relevant assessment of ROI within a diverse multi project grant portfolio.

- Comment (1b): Transparently report your delimitation process

Response: We thank the reviewer for this comment, and as mentioned in response to earlier Comment (1a) we have now added paragraphs on page 9-10 line 256-288 addressing the reviewer’s comment in the revised manuscript explaining the delimitation process.

The evaluation encountered considerable complexity due to the heterogeneity of the 15 projects, each operating within different organisational, cultural, and system environments and commencing at staggered times during 2022-2023. Additional challenges included the absence of common outcome measures, differing ethics and governance requirements, staffing delays, and coordination demands across multiple international time zones. These limitations could have compromised comparability and rigour if not addressed systematically.

In response, the evaluation team developed a comprehensive and methodologically robust roadmap to ensure integrity and consistency across the Program. This roadmap combined innovative analytical approaches, a co design methodology, and sustained engagement with Movember and all Project teams.

This process resulted in the identification of five consistent mental health and well being indicators - depression and anxiety, trauma, resilience, support, and quality of life, aligned with Movember’s MO5 behaviour objectives. The outcome measures that were considered concurrent or convergent by the expert panel and thereby could be used as a proxy in the multi-layered statistical analysis, efficiency analysis and Social Return on Analysis evaluations. These indicators provided the foundation for synthesising findings across highly diverse Projects and contexts.

The following activities were performed during this co-design and preparation period:

• Establish a data sharing agreement with each of the 15 organisations and confirm each project has ethical approval and consent forms which includes sharing de-identified data with UC.

• Confirm the logic of change (inputs, throughputs, and outputs), target audience/s and sample size for each of the Projects.

• Gather information relevant to the evaluation e.g. intervention mechanisms, time frames, recruitment period.

• Establish domains of inputs, throughputs and outputs across all Projects and the relevant parameters of some domains (e.g. frequency of best practice range) with a panel of Project’s representatives and Movember.

• Identify the set of outcome measures adopted by the different projects in each indicator, acknowledge the common outcome measure for each indicator for evaluation, using a novel scientific approach of calibration with an expert panel of clinicians and researchers to determine the level of concurrence and convergence on the properties of each outcome measure for a specific indicator.

• Affirm time points that the Projects will be collecting data, the minimum participant numbers for the various analysis methodology (e.g. statistical analysis, SROI)

• Collaborate with the Projects to support their data collection, pro-vide support or advice as needed, develop systems for secure data transfer within the seven country jurisdictions.

Despite the significant variability across countries, project designs, and implementation realities, this evaluation framework enabled the generation of meaningful, comparable, and policy relevant insights. The co designed, collaborative approach ensured that the evaluation not only met scientific standards but also contributed to collective organisational learning and practical guidance for future investments in VFR mental health.

- Comment (1c): Acknowledge if you took a pragmatic approach based on data availability

Response: We thank the reviewer for this comment. We indeed adopted an ecosystem based, real world pragmatic approach that recognises context and addresses the complexities inherent in evaluating a multi step, mixed methods, and highly complex evaluation process. We have addressed this point in our responses to the Reviewer’s earlier comments and have incorporated corresponding text into the revised manuscript on pages 7-8 and 9-10 to clarify this overarching approach.

- Comment (1d): Justify why broader social value was excluded despite being mentioned in your rationale

Response: We thank the reviewer for this comment and appreciate the concern regarding the inability to estimate an SROI within this evaluation. As explained on pages 6-7 (lines 177-185), SROI relies heavily on subjective assumptions, stakeholder input, and the use of proxy monetary values. While these features can be useful for capturing rich, context specific impacts at an individual project level, they also introduce considerable variability. The absence of a standardised framework for valuing health outcomes and measuring resource use increases the risk of project specific bias. Moreover, monetising non market outcomes can oversimplify complex social impacts, making it difficult to generate comparable results across multiple projects—an essential requirement for evaluating the Movember VFR Program as a whole.

� Reviewer’s Comment (2):

Cross-Program Application and Risk of Misvaluation: The reviewer raises serious concerns about applying a uniform outcome measure across heterogeneous programs. You must:

- Comment (2a): Provide detailed descriptions of each program's specific aims and intended outcomes

- Comment (2b): Clearly identify which programs actually targeted depression/anxiety versus other outcomes

Response: We thank the reviewer for this important comment. We agree that addressing it will strengthen the revised manuscript. As noted in our earlier response, this manuscript is part of a broader, multi layered evaluation of the Movember and Distinguished Gentleman’s Ride–funded Veteran and First Responders (VFR) Mental Health Program.

The evaluation team identified five domains to assess overall Project and Program performance and enable comparison across heterogeneous initiatives. Funder Return on Investment is one of these domains; the others are Complexity, Implementation Processes, Outcomes, and Efficiency.

We appreciate the reviewer’s concerns regarding the use of uniform outcome measures across diverse programs. However, the identification of domains, indicators, and outcome measures involved multiple stages of innovative analytical work, a rigorous co design process, and sustained engagement with Movember and all Project teams. The step by step protocol and multi step comparative evaluation have been published elsewhere (Lukersmith et al., 2025a, 2025b).

To address the reviewer’s comment, we first note that the initial and most relevant step in our process was the identification of evaluation indicators.

The domains were evaluated using a co designed indicator set based on a Logic of Change framework (inputs, throughputs, and outputs, including participant outcomes). A multi step process was used to develop indicators for the Projects and Program evaluations and to create a common set across all 15 Projects. Initial indicators were drafted by the evaluation team (authors) using the Logic of Change model.

An external peer review process was then undertaken to refine and finalise the projects targeted outcome indicators. The Indicators Expert Panel (n=11), comprising evaluation experts, Movember representatives, team members, and external advisors (such as a statistician, evaluation specialist, and mental health expert), began its work in 2021. The panel met twice, supported by follow up email consultations for members unable to attend. Through this consensus process, the initial indicator set was revised and reduced to its final form. The five consistent mental health and well being indicators include depression and anxiety, trauma, resilience, support, and quality of life.

The second relevant step in this process was identifying and acknowledging the heterogeneity in project participant outcome measures.

Project heterogeneity (e.g., target groups, intervention types) led to wide variation in outcome measures. Although Movember and the evaluation team encouraged two common outcome measures (GAD-7 and PHQ-9 in the space of depression and anxiety that can be treated and translated into overall health related quality of life (HQoL)) to enable cross Project comparison, this was not feasible due to differing project aims, concerns about survey burden and ethics, and timing constraints. Consequently, the projects used 120 different outcome measures.

To address this, the evaluation team applied a calibration approach using clinical experts to assess convergence between measures. The process identified six commonly measured indicators: depression, anxiety, trauma related stress, resilience, support, and quality of life. Across these, Projects used 39 different outcome measures, and a “common comparator” was selected for each indicator based on a similar External Expert panel peer review process described earlier.

The evaluation team reviewed all outcome measures for constructs, psychometrics, and validity, comparing each with the common comparator (e.g., Patient Health Questionnaire for Depression symptoms (PHQ-2) vs. Center for Epidemiologic Studies Depression Scale (CESD R), Beck Depression Inventory II for Depression symptom (BDI_II), Patient Health Questionnaire for Depression symptoms (PHQ 4)). The expert panel (n=14) assessed concurrence and convergence using nominal group methods over three meetings (Aug-Sept 2023). This enabled evaluation team to compare outcomes across more projects, though some indicators had data from only up to eight projects.

- Comment (2c): Explicitly discuss the risk of undervaluing programs by measuring inappropriate outcomes

Response: In addressing the reviewer’s comment, we emphasise that although FROI was one domain within the broader evaluation, the authors examined all five outcome indicators targeted across the projects—depression and anxiety, trauma related stress, resilience, support, and quality of life. Crucially, several of these indicators (e.g., trauma related stress, resilience, and support) could not be mapped to utility values or converted into Health Related Quality of Life utility values (HRQoL), which are required to calculate a return on investment (ROI) from the funder’s perspective.

For this reason, we selected the PHQ 9 and GAD 7 not only because they were identified as the Common Comparators across the multiple OMs within the depression and anxiety domains, but also because they are widely used, condition specific symptom measures that can be translated to HRQol utilities for economic evaluation (Mukuria et al, 2024), ensuring that programs are not undervalued by relying on only comparable or inappropriate outcome measures.

- Comment (2d): Consider whether all programs should be included in this analysis framework

Response: As stated in the manuscript and we have now explicitly added a sentence to the 4.22 Study Design and Sample Size sub-section (p. 12, lines 335-339), that was earlier in the limitation section, FROI analyses could not be conducted for all 15 eligible projects due to factors such as the absence of a suitable outcome measure or insufficient sample size. This limitation reflects feasibility constraints rather than an undervaluation of these programs. Moreover, although these projects could not be included in the FROI calculation, they were fully evaluated across the other performance domains in the overall evaluation framework.

The use of PHQ 9 and GAD 7 in the FROI analysis was methodologically justified, consistent with best practice, and necessary to ensure valid and comparable economic estimates, without disadvantaging programs for which economic valuation was not feasible.

� Reviewer’ Comment (3):

Utility and Actionable Insights: Your manuscript currently lacks clear practical implications. Yo

Attachment Submitted filename: Response to Reviewers Comments.docx

# Peer review: Decision Letter 1

**DOI:** 10.1371/journal.pone.0353179.r003

**Authors:** Kuo-Cherh Huang

4 Jun 2026

PONE-D-25-50425R1

Valuing Impact: Estimating Return on Investment of Mental Health and Wellbeing Projects for Veterans and First Responders

PLOS One

Dear Dr. Mohanty,

Thank you for submitting your manuscript to PLOS ONE. After careful consideration, we feel that it has merit but does not fully meet PLOS ONE’s publication criteria as it currently stands. Therefore, we invite you to submit a revised version of the manuscript that addresses the points raised during the review process.

Please submit your revised manuscript by Jul 19 2026 11:59PM. If you will need more time than this to complete your revisions, please reply to this message or contact the journal office at plosone@plos.org. When you're ready to submit your revision, log on to https://www.editorialmanager.com/pone/ and select the 'Submissions Needing Revision' folder to locate your manuscript file.

Please include the following items when submitting your revised manuscript:

- A letter that responds to each point raised by the academic editor and reviewer(s). You should upload this letter as a separate file labeled 'Response to Reviewers'.
- A marked-up copy of your manuscript that highlights changes made to the original version. You should upload this as a separate file labeled 'Revised Manuscript with Track Changes'.
- An unmarked version of your revised paper without tracked changes. You should upload this as a separate file labeled 'Manuscript'.

If you would like to make changes to your financial disclosure, please include your updated statement in your cover letter. Guidelines for resubmitting your figure files are available below the reviewer comments at the end of this letter.

If applicable, we recommend that you deposit your laboratory protocols in protocols.io to enhance the reproducibility of your results. Protocols.io assigns your protocol its own identifier (DOI) so that it can be cited independently in the future. For instructions see: https://journals.plos.org/plosone/s/submission-guidelines#loc-laboratory-protocols. Additionally, PLOS ONE offers an option for publishing peer-reviewed Lab Protocol articles, which describe protocols hosted on protocols.io. Read more information on sharing protocols at https://plos.org/protocols?utm_medium=editorial-email&utm_source=authorletters&utm_campaign=protocols.

As the corresponding author, your ORCID iD is verified in the submission system and will appear in the published article. PLOS supports the use of ORCID, and we encourage all coauthors to register for an ORCID iD and use it as well. Please encourage your coauthors to verify their ORCID iD within the submission system before final acceptance, as unverified ORCID iDs will not appear in the published article. *Only*  the individual author can complete the verification step; PLOS staff *cannot*  verify ORCID iDs on behalf of authors.

We look forward to receiving your revised manuscript.

Kind regards,

Kuo-Cherh Huang

Academic Editor

PLOS One

**Journal Requirements:**

1. If the reviewer comments include a recommendation to cite specific previously published works, please review and evaluate these publications to determine whether they are relevant and should be cited. There is no requirement to cite these works unless the editor has indicated otherwise.

2. Please review your reference list to ensure that it is complete and correct. If you have cited papers that have been retracted, please include the rationale for doing so in the manuscript text, or remove these references and replace them with relevant current references. Any changes to the reference list should be mentioned in the rebuttal letter that accompanies your revised manuscript. If you need to cite a retracted article, indicate the article’s retracted status in the References list and also include a citation and full reference for the retraction notice.

**Additional Editor Comments:**

Dear Dr. Mohanty,

Thank you for submitting your revised manuscript to PLOS ONE. Reviewer 1 had nicely provided several additional suggestions to polish your work, which are relatively minor, as noted below. Thank you.

Kuo-Cherh Huang

Academic Editor

[Note: HTML markup is below. Please do not edit.]

Reviewers' comments:

Reviewer's Responses to Questions

**Comments to the Author**

1. If the authors have adequately addressed your comments raised in a previous round of review and you feel that this manuscript is now acceptable for publication, you may indicate that here to bypass the “Comments to the Author” section, enter your conflict of interest statement in the “Confidential to Editor” section, and submit your "Accept" recommendation.

Reviewer #1: (No Response)

**********

2. Is the manuscript technically sound, and do the data support the conclusions?

The manuscript must describe a technically sound piece of scientific research with data that supports the conclusions. Experiments must have been conducted rigorously, with appropriate controls, replication, and sample sizes. The conclusions must be drawn appropriately based on the data presented.

Reviewer #1: Yes

**********

3. Has the statistical analysis been performed appropriately and rigorously?

Reviewer #1: Yes

**********

4. Have the authors made all data underlying the findings in their manuscript fully available?

The PLOS Data policy requires authors to make all data underlying the findings described in their manuscript fully available without restriction, with rare exception (please refer to the Data Availability Statement in the manuscript PDF file). The data should be provided as part of the manuscript or its supporting information, or deposited to a public repository. For example, in addition to summary statistics, the data points behind means, medians and variance measures should be available. If there are restrictions on publicly sharing data—e.g. participant privacy or use of data from a third party—those must be specified.

Reviewer #1: No

**********

5. Is the manuscript presented in an intelligible fashion and written in standard English?

PLOS ONE does not copyedit accepted manuscripts, so the language in submitted articles must be clear, correct, and unambiguous. Any typographical or grammatical errors should be corrected at revision, so please note any specific errors here.

Reviewer #1: Yes

**********

6. Review Comments to the Author

Please use the space provided to explain your answers to the questions above. You may also include additional comments for the author, including concerns about dual publication, research ethics, or publication ethics. (Please upload your review as an attachment if it exceeds 20,000 characters)

Reviewer #1: Thank you for addressing the reviewer comments thoughtfully. They have addressed my comments substantively.

I found some minor issues that you should address before finalising the manuscript. They are:

Page 2 (abstract), line 77: given you list several reasons, 'contribute' should be plural

Page 6 line 165: 'monetise' should be monetises (to match 'converts')

Page 7, line 185: suggest 'analysis of the 2021 Movember VFR...' which makes it clear that is the funding/granting date, rather than the date of your analysis.

Page 8 line 217: teams with the full protocol (add 'the)

Page 9 line 260: are all 108 outcomes participant outcomes? or were there some program outcomes here? There can be program outcomes that are not participant outcomes - so please check.

Page 10 line 268: and are published elsewhere (add 'are')

Page 11 line 295. Sentence starting with 'However...' does not make sense. Please check and clarify.

Page 10 line 315: 'change is utility' should be 'change in utility', and there is 'ad' at the end of the sentence before the struck out text

Page 12 line 337: should clarify were only able to include 11 projects 'in the analysis'

Page 16 line 421: substitute 'get' with achieve

Page 22 lie 314: how many projects excluded in the first sensitivity approach? you note 2 projects in the second approach.

Page 25 line 410: sentence starting 'Therefore, we encourage..' does not make sense. Please check and clarify.

**********

7. PLOS authors have the option to publish the peer review history of their article (what does this mean?). If published, this will include your full peer review and any attached files.

If you choose “no”, your identity will remain anonymous but your review may still be made public.

**Do you want your identity to be public for this peer review?**  For information about this choice, including consent withdrawal, please see our Privacy Policy.

Reviewer #1: No

**********

[NOTE: If reviewer comments were submitted as an attachment file, they will be attached to this email and accessible via the submission site. Please log into your account, locate the manuscript record, and check for the action link "View Attachments". If this link does not appear, there are no attachment files.]

To ensure your figures meet our technical requirements, please review our figure guidelines: https://journals.plos.org/plosone/s/figures

You may also use PLOS’s free figure tool, NAAS, to help you prepare publication quality figures: https://journals.plos.org/plosone/s/figures#loc-tools-for-figure-preparation.

NAAS will assess whether your figures meet our technical requirements by comparing each figure against our figure specifications.

# Author comment: Author response to Decision Letter 2

**DOI:** 10.1371/journal.pone.0353179.r004

18 Jun 2026

We thank the Reviewer for their thoughtful feedback and the editor for providing the opportunity to revise our manuscript. We believe that addressing these comments has significantly enhanced the presentation of the revised manuscript. Please note that all page and line numbers referenced in this document correspond to the Revised manuscript with track changes enabled.

� Reviewer #1: Thank you for addressing the reviewer comments thoughtfully. They have addressed my comments substantively.

Author Response: We thank the reviewer for this encouraging comment.

� Reviewer #1: I found some minor issues that you should address before finalising the manuscript. They are:

Page 2 (abstract), line 77: given you list several reasons, 'contribute' should be plural

Author Response: This is now edited in the revised draft.

� Page 6 line 165: 'monetise' should be monetises (to match 'converts')

Author Response: We thank the reviewer for identifying this typographical error. It has now been corrected in the revised manuscript on page#6 line#163.

� Page 7, line 185: suggest 'analysis of the 2021 Movember VFR...' which makes it clear that is the funding/granting date, rather than the date of your analysis.

Author Response: We thank the reviewer for identifying this discrepancy in dates. This sentence has now been edited in the revised manuscript. The revised text on page#6 line#183-184 now reads as “we conduct an economic analysis of the ROI for the funder as part of a broader impact analysis of the 2021 Movember VFR Program with final data collected from each project by March 2024”.

� Page 8 line 217: teams with the full protocol (add 'the)

Author Response: We thank the reviewer for identifying this typographical error. It has now been corrected in the revised manuscript on page#7 line#198.

� Page 9 line 260: are all 108 outcomes participant outcomes? or were there some program outcomes here? There can be program outcomes that are not participant outcomes - so please check.

Author Response: This text has now been edited in the revised manuscript on page#8 line#235. Just to confirm, yes, there were120 patient reported outcome measures (PROMs)used across 15 projects for different mental health outcomes.

� Page 10 line 268: and are published elsewhere (add 'are')

Author Response: We thank the reviewer for identifying this typographical error. It has now been corrected in the revised manuscript on page#9 line#246.

� Page 11 line 295. Sentence starting with 'However...' does not make sense. Please check and clarify.

Author Response: We thank the reviewer for this comment. It has now been edited in the revised manuscript on page#10 line#270.

� Page 10 line 315: 'change is utility' should be 'change in utility', and there is 'ad' at the end of the sentence before the struck out text

Author Response: We thank the reviewer for identifying this typographical error. It has now been edited in the revised manuscript on page#10 line#290.

� Page 12 line 337: should clarify were only able to include 11 projects 'in the analysis'

Author Response: We thank the reviewer for identifying this missing phrase. It has now been edited in the revised manuscript on page#11 line#311.

� Page 16 line 421: substitute 'get' with achieve

Author Response: We thank the reviewer for this comment. It has now been edited in the revised manuscript on page#15 line#379.

� Page 22 line 314: how many projects excluded in the first sensitivity approach? you note 2 projects in the second approach.

Author Response: We thank the reviewer for this comment. It has now been edited in the revised manuscript on page#20 line#311-313.

� Page 25 line 410: sentence starting 'Therefore, we encourage..' does not make sense. Please check and clarify.

Author Response: We thank the reviewer for this comment. This text has now been edited in the revised manuscript on page#23 line#404-405.

Attachment Submitted filename: Response to Reviewer.docx

# Editor report: Decision Letter 2

**DOI:** 10.1371/journal.pone.0353179.r005

**Authors:** Kuo-Cherh Huang

21 Jun 2026

Valuing Impact: Estimating Return on Investment of Mental Health and Wellbeing Projects for Veterans and First Responders

PONE-D-25-50425R2

Dear Dr. Mohanty,

We’re pleased to inform you that your manuscript has been judged scientifically suitable for publication and will be formally accepted for publication once it meets all outstanding technical requirements.

Within one week, you’ll receive an e-mail detailing the required amendments. When these have been addressed, you’ll receive a formal acceptance letter and your manuscript will be scheduled for publication.

An invoice will be generated when your article is formally accepted. Please note, if your institution has a publishing partnership with PLOS and your article meets the relevant criteria, all or part of your publication costs will be covered. Please make sure your user information is up-to-date by logging into Editorial Manager at Editorial Manager® and clicking the ‘Update My Information' link at the top of the page. For questions related to billing, please contact billing support.

If your institution or institutions have a press office, please notify them about your upcoming paper to help maximize its impact. If they’ll be preparing press materials, please inform our press team as soon as possible -- no later than 48 hours after receiving the formal acceptance. Your manuscript will remain under strict press embargo until 2 pm Eastern Time on the date of publication. For more information, please contact onepress@plos.org.

Kind regards,

Kuo-Cherh Huang

Academic Editor

PLOS One

Additional Editor Comments (optional):

Reviewers' comments:

# Editor report: Acceptance letter

**DOI:** 10.1371/journal.pone.0353179.r006

**Authors:** Kuo-Cherh Huang

PONE-D-25-50425R2

PLOS One

Dear Dr. Mohanty,

I'm pleased to inform you that your manuscript has been deemed suitable for publication in PLOS One. Congratulations! Your manuscript is now being handed over to our production team.

At this stage, our production department will prepare your paper for publication. This includes ensuring the following:

* All references, tables, and figures are properly cited

* All relevant supporting information is included in the manuscript submission,

* There are no issues that prevent the paper from being properly typeset

You will receive further instructions from the production team, including instructions on how to review your proof when it is ready. Please keep in mind that we are working through a large volume of accepted articles, so please give us a few days to review your paper and let you know the next and final steps.

Lastly, if your institution or institutions have a press office, please let them know about your upcoming paper now to help maximize its impact. If they'll be preparing press materials, please inform our press team within the next 48 hours. Your manuscript will remain under strict press embargo until 2 pm Eastern Time on the date of publication. For more information, please contact onepress@plos.org.

You will receive an invoice from PLOS for your publication fee after your manuscript has reached the completed accept phase. If you receive an email requesting payment before acceptance or for any other service, this may be a phishing scheme. Learn how to identify phishing emails and protect your accounts at https://explore.plos.org/phishing.

If we can help with anything else, please email us at customercare@plos.org.

Thank you for submitting your work to PLOS ONE and supporting open access.

Kind regards,

PLOS ONE Editorial Office Staff

on behalf of

Dr. Kuo-Cherh Huang

Academic Editor

PLOS One
